# Polyoxometalates enable cellular uptake of the actin-binding toxin phalloidin

**DOI:** 10.1039/d6tb00818f

**Published:** 2026-08-04

**Authors:** Nadiia Gumerova, Janice Bergen, Joshua Rieger, Giorgia Del Favero, Annette Rompel

**Affiliations:** a Universität Wien, Fakultät für Chemie, Institut für Biophysikalische Chemie Josef-Holaubek-Platz 2 1090 Wien Austria nadiia.gumerova@univie.ac.at annette.rompel@univie.ac.at https://www.bpc.univie.ac.at; b Department of Food Chemistry and Toxicology, Faculty of Chemistry, University of Vienna Vienna Austria https://lmc.univie.ac.at; c Core Facility Multimodal Imaging, Faculty of Chemistry, University of Vienna Vienna Austria https://multimodal-imaging.univie.ac.at; d Universität Wien, Fakultät für Chemie, Vienna Doctoral School in Chemistry (DoSChem) Währinger Straße 42 1090 Wien Austria https://doschem.univie.ac.at

## Abstract

Superchaotropic inorganic cluster anions can transport membrane-impermeable biomolecules across lipid bilayers, yet how carrier structure governs delivery efficiency remains unclear. Here we show that two closely related Keggin-type polyoxometalates (POMs)—[SiW_12_O_40_]^4−^ (SiW_12_) and its mono-molybdenum-substituted analogue [SiMoW_11_O_40_]^4−^ (SiMoW_11_)—differ markedly in their efficiency to deliver phalloidin, a rigid membrane-impermeable F-actin probe, into colorectal carcinoma cells. Despite near-identical size, charge, and charge density, SiW_12_ undergoes extensive hydrolysis at pH 7.5 while SiMoW_11_ shows only partial spectral reorganisation indicative of structural rearrangement, as established by ^183^W NMR spectroscopy. The direct quantitative comparison of the two compounds in cell culture medium was not accessible, due to the inherent lack of sensitivity that exists in ^183^W NMR under these conditions. Both POMs are non-toxic up to 100 µM and facilitate phalloidin uptake and F-actin staining in two human colorectal carcinoma cell lines (HCT116 and HT29), with SiMoW_11_ delivering phalloidin more effectively than SiW_12_ at equivalent carrier and cargo concentrations.

## Abstract 2

Keggin-type polyoxometalates deliver membrane-impermeable phalloidin into living colorectal carcinoma cells. Single-atom molybdenum substitution improves intracellular delivery efficiency.

## Graphical abstract

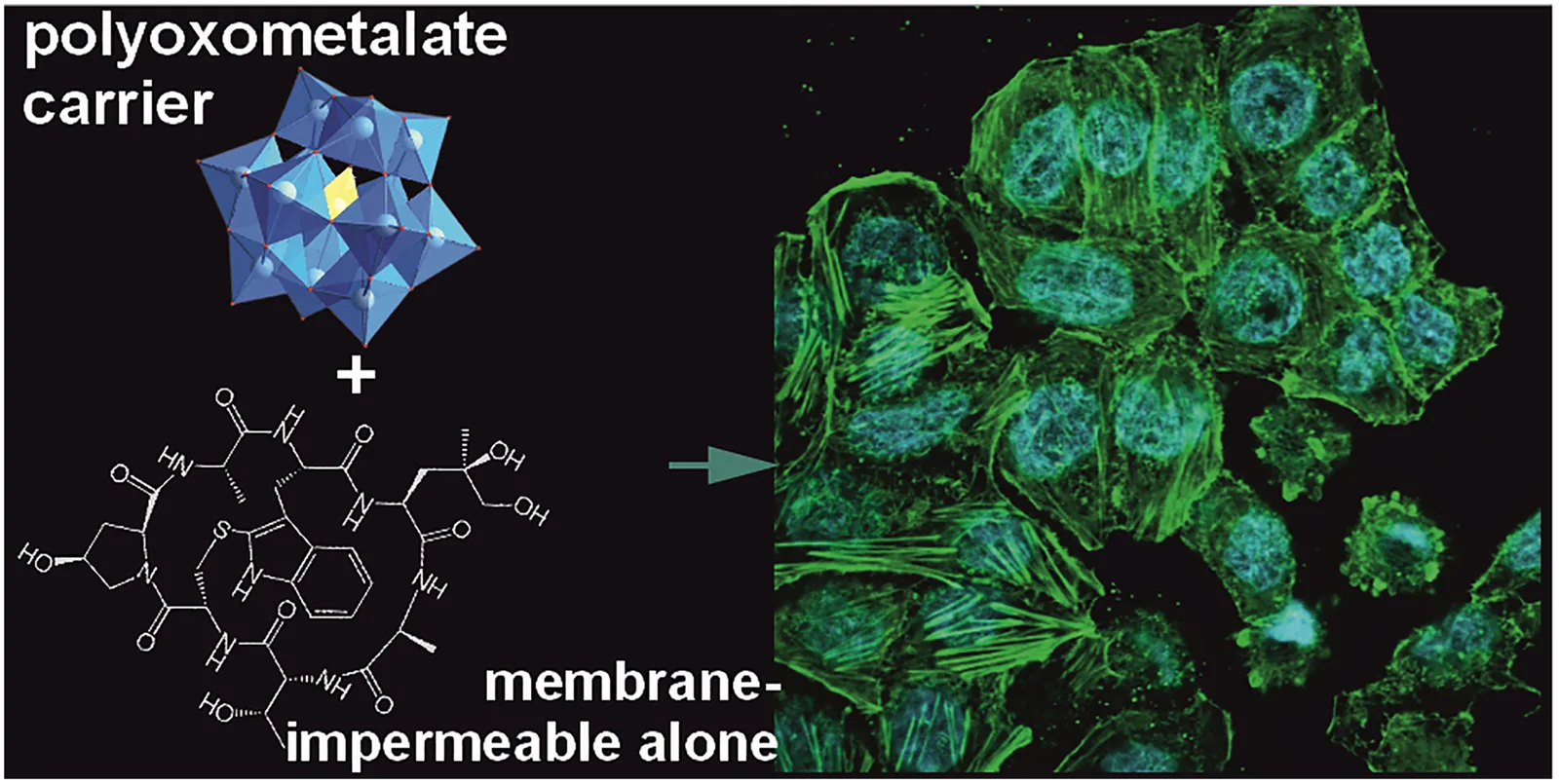

## Introduction

**Fig. 1:**
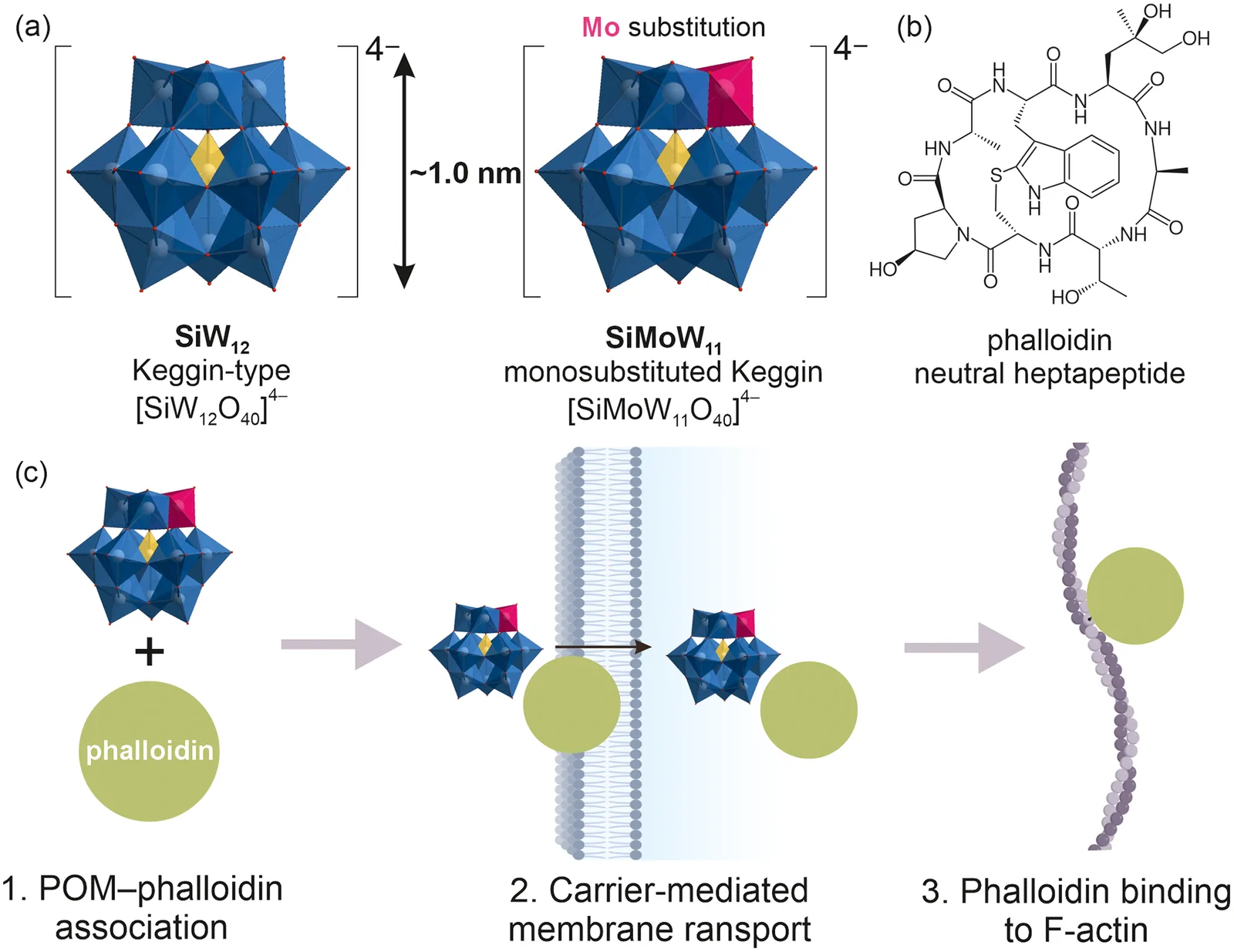
POMs as superchaotropic carriers. (a) Structures of the Keggin-type POMs SiW_12_ ([SiW_12_O_40_]^4−^) and SiMoW_11_ ([SiMoW_11_O_40_]^4−^). (b) Molecular structure of phalloidin. (c) Schematic representation of the proposed transport process: (1) POM–phalloidin association in solution through non-covalent interactions; (2) carrier-mediated transport of the complex across the lipid bilayer; and (3) intracellular binding of phalloidin to F-actin, enabling cytoskeletal staining. Color code: {WO_6_}, blue; {MoO_6_}, magenta; {SiO_4_}, yellow; O, red.

The controlled transport of polar and membrane-impermeable biomolecules across lipid bilayers remains a central challenge in chemical biology, biotechnology, and materials science.^1,2^ While nanoparticles,^3^ liposomal systems,^4^ and cell-penetrating peptides^5^ have enabled significant progress, their performance is often governed by complex supramolecular assemblies, heterogeneous structures, or irreversible membrane disruption.^6^ Superchaotropic^7^ inorganic clusters have recently been shown to act as non-covalent carriers capable of promoting membrane translocation of otherwise impermeable biomolecules.^8–10^ Notably, boron cluster anions enable delivery of diverse hydrophilic cargos, including peptides and the rigid bicyclic toxin phalloidin, without covalent cargo modification.^8^ In parallel, we recently demonstrated that mixed-metal Keggin-type polyoxometalates (POMs, Fig. 1) can function as efficient membrane carriers for cationic peptides in model membranes and mammalian cells, establishing compositionally defined POMs as a new class of molecular transport materials.^9^ These studies^8–10^established functional transport capability but left key mechanistic questions unresolved. In particular, the nature of the solution-phase interaction between inorganic clusters and cargo molecules, and how such interactions translate into membrane activity, has not been directly analyzed.

Phalloidin provides an exceptionally demanding test system for probing inorganic carrier-mediated transport.^8,10^ This rigid bicyclic heptapeptide (Fig. 1b) derived from *Amanita phalloides* binds with high specificity to F-actin and is widely used as a cytoskeletal marker.^11^ However, phalloidin cannot cross intact cell membranes unaided, and its standard application as a fluorescent actin stain therefore requires prior fixation and chemical permeabilization of cells—conditions that preclude live-cell imaging and limit its utility as a dynamic cytoskeletal probe.^12^ Delivery strategies typically require covalent modification with polycationic carriers^12^ or deliberate membrane disruption,^13^ and non-covalent low-molecular-weight transport systems remain rare.^10^ Importantly, phalloidin's structural rigidity, polarity, and a defined intracellular target provide a stringent functional readout: successful delivery requires not only membrane crossing but preservation of biological activity and access to cytosolic F-actin. These features make cytoskeletal fluorescence an easily measurable parameter upon successful delivery, confirming membrane crossing and preservation of cargo bioactivity, while remaining insensitive to the individual mechanistic contributions—solution association, membrane perturbation, and transport efficiency—that collectively determine carrier potency. These are addressed separately through complementary solution-phase and membrane-interaction measurements.

Here, we investigate the polyoxometalate-mediated transport of phalloidin across biological membranes by directly connecting molecular association and cellular uptake. We focus on two closely related Keggin-type polyoxometalates, [SiW_12_O_40_]^4−^ (SiW_12_) (Fig. 1a, left) and the monosubstituted analogue [SiMoW_11_O_40_]^4−^ (SiMoW_11_) (Fig. 1a, right). Both clusters possess nearly identical molecular dimensions (∼1 nm), the same formal charge (4−), and a comparable geometric charge distributions (approximately −0.33 charge per addenda metal center and an estimated surface charge density of ∼0.011*e* Å^−2^). Based on these similarities, both anions are expected to exhibit comparable solution stability and membrane transport behavior. However, in our previous work^9^ we observed that SiW_12_ undergoes more pronounced hydrolysis under physiological conditions and displays lower membrane transport activity in model vesicle systems compared to its monosubstituted counterpart SiMoW_11_. This observation suggests that even minimal structural modification within an otherwise identical cluster framework can significantly influence its physicochemical behavior. From a chemical perspective, this pair of closely related Keggin anions therefore provides an ideal system to investigate how single-metal substitution modulates cluster stability, membrane transport activity, and biological response. By linking molecular association to membrane interaction and biological transport, this study provides mechanistic insight into how discrete inorganic clusters can function as adaptive molecular carriers for rigid biomolecular cargo. The superchaotropic membrane adsorption mechanism established for SiMoW_11_ in model membrane systems^9^ provides a working mechanistic framework for the cellular transport observed here, while the precise internalization pathway remains an open question and is the subject of ongoing investigation.

The solution speciation of SiW_12_ and SiMoW_11_ under conditions of increasing biological complexity was characterized by ^183^W NMR spectroscopy, the method of choice for direct, quantitative monitoring of polyoxotungstate speciation in solution.^14^ For a substituted *α*-Keggin anion of the [SiMoW_11_O_40_]^4−^ type, replacement of one framework W by Mo breaks the *T*_d_ symmetry of the parent [SiW_12_O_40_]^4−^ cluster and renders the eleven remaining tungsten centers chemically inequivalent, giving rise to a characteristic six-signal ^183^W NMR fingerprint that serves as a direct reporter of structural integrity.

**Fig. 2:**
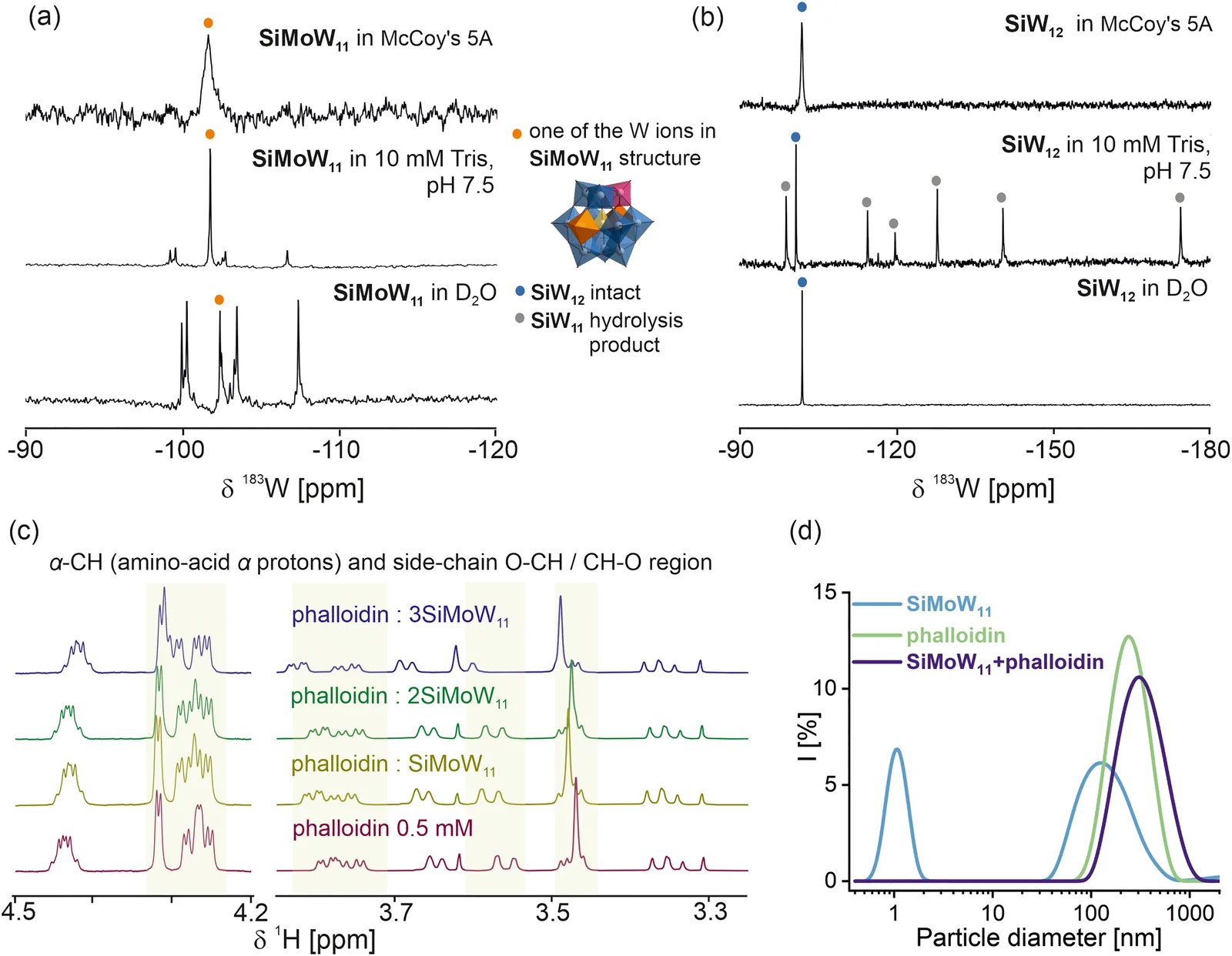
Solution stability and interaction of Keggin-type polyoxometalates with phalloidin. (a) ^183^W NMR spectra of SiMoW_11_ (60 mg mL^−1^) recorded in different media: D_2_O, 10 mM Tris buffer (pH 7.5), and McCoy's 5A cell culture medium. (b) ^183^W NMR spectra of SiW_12_ (60 mg mL^−1^) in D_2_O, 10 mM Tris buffer (pH 7.5), and in McCoy's 5A. Blue markers indicate the intact SiW_12_ Keggin anion, while grey markers correspond to hydrolysis products (6 signals belong to [SiW_11_O_39_]^8−^ type species). (c) ^1^H NMR spectra of phalloidin (0.5 mM) in the absence and presence of increasing amounts of SiMoW_11_ (phalloidin : POM ratios 1 : 1–1 : 3). Phalloidin was dissolved in DMSO-d_6_, the POM stock solution was prepared in D_2_O with 150 mM NaCl; the percentage of DMSO-d_6_ was 4% in each tube. Highlighted regions correspond to the *α*-CH protons of amino acid residues (Fig. 1b) and the side-chain O–CH/CH–O (see structure of phalloidin in Fig. 1b) resonances, where chemical shift perturbations are observed upon addition of the POM. (d) Dynamic light scattering (DLS) analysis of SiMoW_11_ (5 mg mL^−1^), phalloidin, and their mixture, showing the particle size distributions and indicating the formation of larger assemblies in the presence of both components. Color code: {WO_6_}, blue; {MoO_6_}, magenta; {SiO_4_}, yellow; O, red.

In pure water, both SiW_12_ and SiMoW_11_ are fully intact: each compound yielded a clean ^183^W NMR spectrum consistent with the undegraded Keggin framework, with no evidence of hydrolysis products (Fig. 2a). For SiW_12_, the single resonance expected for [SiW_12_O_40_]^4−^ was observed with no additional signals; for SiMoW_11_, the expected pattern of symmetry-inequivalent tungsten environments arising from substitution of one W by Mo—in which the remaining eleven W centers split into chemically distinct sets—was fully resolved, confirming the structural integrity of the substituted cluster. The stability changed substantially upon dissolution in 10 mM Tris–HCl pH 7.5, conditions designed to approximate the buffering capacity of biological media while minimizing ionic complexity. Under these conditions, SiW_12_ underwent extensive hydrolysis: approximately 50% of the compound was converted to the monolacunary species [SiW_11_O_39_]^8−^ (Scheme S1), as evidenced by the emergence of the characteristic ^183^W signals of the *C*_s_-symmetric lacunary anion alongside the diminished resonance of the intact cluster (Fig. 2b). Notably, SiMoW_11_ also showed alterations in its ^183^W signal pattern upon dissolution in 10 mM Tris–HCl pH 7.5, with the weaker resonances arising from tungsten centers positioned closest to the Mo substitution site showing preferential intensity loss, consistent with their greater susceptibility to hydrolytic attack. The dominant signal, corresponding to the most geometrically remote and hydrolytically robust W environments, was retained, although the overall extent of speciation redistribution, which may include partial conversion to SiW_12_, was qualitatively less pronounced than that observed for SiW_12_. This differential stability directly demonstrates that single-ion Mo substitution confers measurable resistance to hydrolysis relative to the all-tungsten parent. This can be rationalized on electronic grounds: Mo^6+^ is more polarizable than W^6+^ and engages in stronger π-backbonding with the bridging oxo ligands, redistributing electron density across the Mo–O–W bridges and reducing the susceptibility of framework oxygen ions to nucleophilic attack by water. As a consequence, the activation barrier for the initial hydrolytic step—loss of a framework metal center to yield the lacunary species—is raised in SiMoW_11_ relative to SiW_12_, resulting in a cluster that retains greater structural integrity under the near-physiological buffered conditions employed here.^15,16^

The complexity of speciation increased further upon dissolution in McCoy's 5A cell culture medium (containing inorganic salts, amino acids, glucose, and vitamins at physiological ionic strength, pH 7.4, full composition provided in the SI, Section S1).^17^ The rich and variable ionic composition of this medium creates a highly competitive ionic environment that both screens the SiMoW_11_ charge and promotes competing ligand exchange at labile metal centers. Under these conditions, the ^183^W NMR spectra of both compounds became substantially simplified: only the most intense resonance remained clearly detectable for SiMoW_11_, with weaker signals falling below the signal-to-noise threshold accessible within a practical acquisition time of ∼60 h (Fig. 2a). This spectral collapse does not necessarily indicate complete decomposition of the cluster; rather, it reflects signal broadening arising from ion pairing with medium components, particularly divalent cations (Ca^2+^, Mg^2+^), phosphate, and amino acids, which increase the rotational correlation time of the POM and enhance nuclear relaxation. This effect is compounded by the inherent insensitivity of ^183^W NMR (natural abundance 14.3%, receptivity ∼0.06% relative to ^1^H), which limits signal detection under the dilute and compositionally complex conditions of cell culture media. The two POMs also differed in their colloidal behavior in McCoy's 5A medium: dissolution of SiMoW_11_ produced visible turbidity that progressed to precipitation over the course of the 60 h measurement period, whereas SiW_12_ remained visually clear under the same conditions. This difference in colloidal stability directly accounts for the near-noise signal intensity observed for SiMoW_11_ in McCoy's 5A medium (Fig. 2a), as removal of the cluster from solution by precipitation reduces the effective concentration available for detection. We note that the POM concentrations required for ^183^W NMR significantly exceed those used in the cellular uptake experiments (≤50 µM), where no precipitation was observed for either compound, and the NMR results in McCoy's medium should therefore be interpreted in the context of this concentration difference. Notably, for both SiW_12_ (Fig. 2b) and SiMoW_11_ (Fig. 2a), a dominant ^183^W resonance remains detectable in McCoy's 5A medium, suggesting that a fraction of each cluster persists in a defined structural form under cell culture conditions, although the spectral simplification prevents any quantitative assessment of speciation or comparison between the two compounds in this matrix.

The interaction between phalloidin and SiMoW_11_ was first examined by ^1^H NMR spectroscopy (Fig. 2c and Fig. S7–S11). Upon addition of up to 3 equivalents of the SiMoW_11_, only small but systematic chemical-shift perturbations were observed in the aromatic region of phalloidin (Fig. S11), while the 3.3–4.5 ppm region showed somewhat more pronounced changes in peak positions and line shapes (Fig. 2c). However, no new well-resolved set of signals and no strong, consistent broadening characteristic of a discrete tight complex were detected. The pH of the solutions was monitored throughout the titration and remained within a narrow range of 4.8–5.0 across all SiMoW_11_ concentrations employed. A variation of 0.1–0.2 pH units is insufficient to induce protonation state changes in phalloidin's ionizable groups—the phenolic hydroxyl (p*K*_a_ ∼ 9.5), indole nitrogen (p*K*_a_ > 14), and carboxylic acid residues (p*K*_a_ ∼ 2.5–3.5) are all fully protonated or deprotonated across this narrow range and pH effects can therefore be excluded as the origin of the observed chemical shift perturbations. Overall, the NMR data point to a weak interaction in fast exchange on the NMR timescale rather than formation of a well-defined 1 : 1 molecular adduct. To directly probe whether a thermodynamically defined binding event occurs, isothermal titration calorimetry (ITC) was performed in 10 mM Tris-HCl pH 7.5. No detectable heat of interaction was observed at micromolar concentrations, confirming the absence of a stoichiometric POM–phalloidin complex under these conditions (Fig. S12). While phalloidin carries no net charge at physiological pH and **SiMoW_11_** bears a formal 4− charge, the absence of classical electrostatic attraction does not preclude association: superchaotropic anions are known to interact with neutral molecules through a combination of weak electrostatic polarization, hydrophobic contacts, and ion-induced dipole interactions at the cluster surface. We note that an ITC signal for boron cluster–phalloidin association was reported by Salluce *et al.*^10^ at millimolar carrier concentrations, conditions under which such weak interactions become calorimetrically detectable. The absence of a signal in our experiments at micromolar concentrations is therefore consistent with an interaction of comparably weak affinity rather than indicative of no association.

To further probe the system on the colloidal scale, dynamic light scattering (DLS) measurements were performed. The SiMoW_11_ solution showed a small-size population around 1 nm, consistent with molecularly dispersed species, together with a minor larger population around 100 nm (Fig. 2d). Phalloidin alone gave a dominant signal around 300 nm, indicating that under these conditions it does not exist exclusively as monomeric molecules but forms larger aggregates or colloidal assemblies. Upon mixing phalloidin with SiMoW_11_, its ∼1 nm population was no longer observed, and the DLS signal became dominated by particles around 350 nm (Fig. 2d). Taken together, these results suggest that phalloidin and SiMoW_11_ do interact, but the interaction is governed primarily by formation of larger supramolecular co-assemblies or aggregates rather than by a strong, discrete molecular complex – a mode of association consistent with the loose, non-stoichiometric interaction reported for boron cluster carriers with phalloidin under analogous conditions.^8^

Keeping in mind potential future applications in *e.g.* cancer treatment, SiW_12_ and SiMoW_11_ were evaluated for the capacity to facilitate the intracellular delivery of membrane-impermeable probes in two well-established human colorectal carcinoma (CRC) cell lines, namely, HCT116 and HT29. Colorectal cancer represents one of the most prevalent malignancies worldwide,^18^ and these two lines differ substantially in their molecular, phenotypic, and membrane-compositional characteristics. HCT116 is a microsatellite-unstable (MSI-H), KRAS-mutant (G13D) line with a relatively uniform epithelial morphology and a well-defined cortical actin network. It proliferates rapidly and maintains a plasma membrane rich in phosphatidylcholine and phosphatidylethanolamine with a moderate cholesterol content, resulting in a fluid, highly dynamic membrane that supports active macropinocytosis and clathrin-mediated endocytosis.^19,20^ HT29, by contrast, is a microsatellite-stable (MSS) line harboring a BRAF^V600E^ mutation, which undergoes partial enterocytic differentiation, producing a thick glycocalyx of heavily O-glycosylated mucins on its apical surface.^19,20^ This glycocalyx presents an additional physicochemical barrier to membrane access, and the HT29 membrane is characterized by higher sphingomyelin and glycosphingolipid content relative to HCT116, conferring greater rigidity and reduced endocytic activity (Table S1). These differences in membrane fluidity, glycocalyx density, and lipid composition could influence the kinetics and extent of POM adsorption and cargo co-transport, making this pair of cell lines particularly informative for benchmarking the superchaotropic carrier activity of the Keggin type SiW_12_ and SiMoW_11_ studied here.

Prior to any co-incubation experiments, the cytotoxicity of the two Keggin-type POMs SiW_12_ and SiMoW_11_ under investigation was determined in both HCT116 and HT29 cells using the CellTiter-Blue (CTB) viability assay.^21^ Critically, neither SiW_12_ nor SiMoW_11_ induced any statistically significant reduction in cell viability at concentrations up to 100 µM in either HCT116 or HT29 cells (Fig. S1), establishing a broad non-cytotoxic concentration range, significantly exceeding that required for phalloidin-TRITC (TRITC = tetramethylrhodamine isothiocyanate) delivery. To further support that the POM–phalloidin combination *per se* does not cause cytotoxic membrane alterations, co-incubation of SiMoW_11_ with 2.5 µM phalloidin-TRITC across the full concentration range used in the uptake experiments (1–100 µM) produced no significant reduction in HCT116 cell viability (Fig. S2), limiting the possibility that lysis-mediated artefacts could contribute to the observed intracellular fluorescence. This confirmed that all subsequent uptake assays were conducted under conditions in which cell viability was preserved, and that any observed fluorescence enhancement is attributable to genuine Keggin POM-facilitated cargo transport rather than to membrane lysis or cytotoxic permeabilization.

**Fig. 3:**
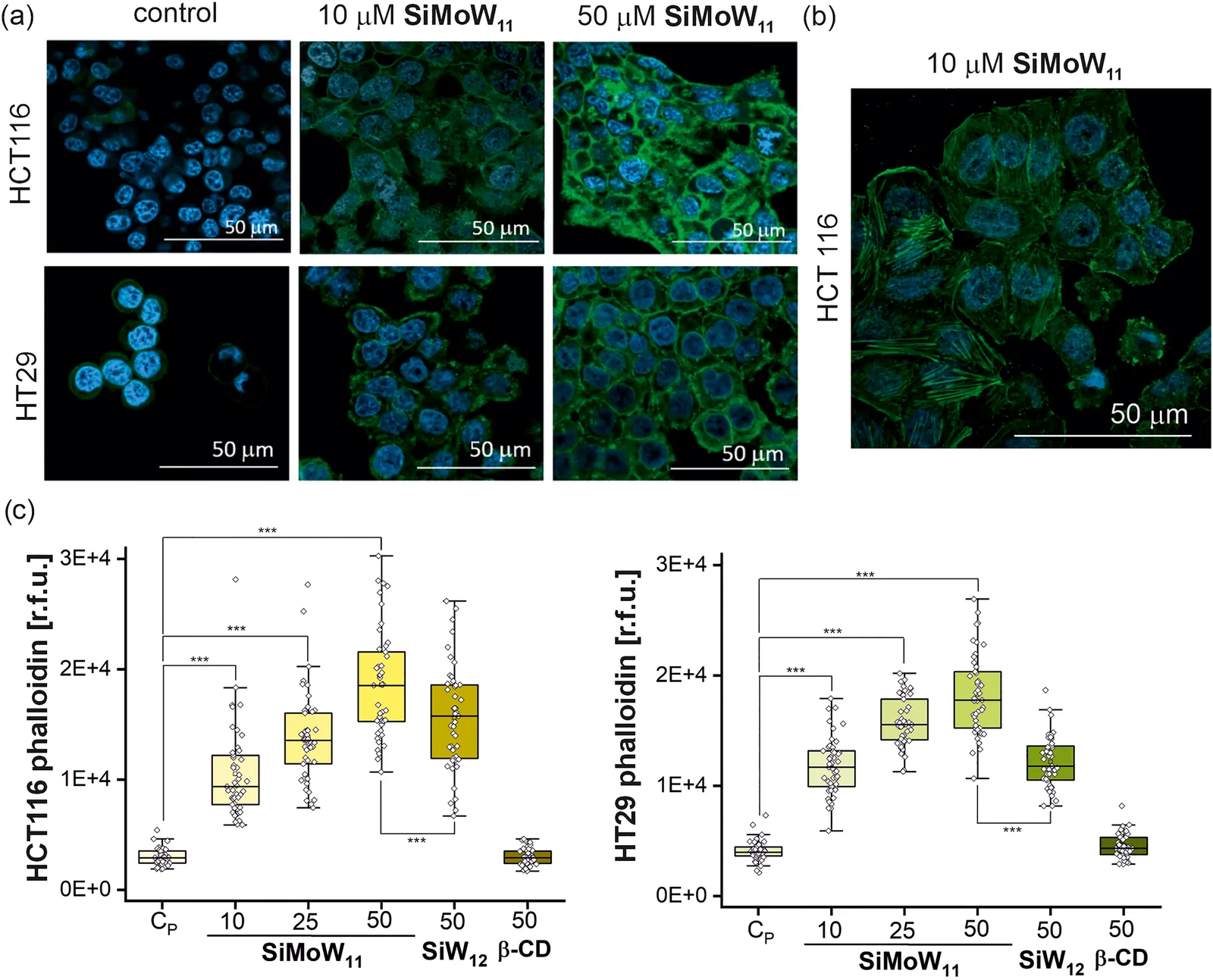
POM-mediated transport of phalloidin-TRITC. (a) and (b) Confocal microscopy images of phalloidin uptake in HCT116 and HT29 cells, cell nuclei are depicted in blue and phalloidin signal in green. (c) Quantification of cellular uptake of 2.5 µM phalloidin-TRITC after 2 h of incubation for controls (C_p_ – cells with phalloidin-TRITC) and substance treatments (10, 25, or 50 µM SiMoW_11_; 50 µM SiW_12_; 50 µM methyl-β-cyclodextrin was added as reference). R.f.u. stands for relative fluorescence units. The significant differences in comparison to the control were determined by applying one-way ANOVA test followed by Fisher's least significant difference (LSD) and marked as ***(*p* < 0.001). Direct comparison between SiMoW_11_ and SiW_12_ at 50 µM was performed using a two-tailed Student's *t*-test; statistically significant differences are indicated as ***(*p* < 0.001). The data were obtained from three independent cell preparations, quantifying *n* ≥ 60 cells for each experimental condition.

Cells were co-incubated with phalloidin-TRITC (2.5 µM) and either SiW_12_ or SiMoW_11_ (at 10, 25 or 50 µM) in serum-free medium for 2 h at 37 °C; control wells received phalloidin-TRITC alone. In the absence of SiW_12_ or SiMoW_11_ no intracellular TRITC fluorescence was detectable in either cell line, which is consistent with the known impermeability of these cells to phalloidin. Co-incubation with SiW_12_ or SiMoW_11_ produced a clear and reproducible enhancement of intracellular fluorescence (Fig. 3 and Fig. S3–S6), with staining patterns characteristic of F-actin (Fig. 3b). SiMoW_11_ consistently outperformed SiW_12_ in terms of phalloidin-TRITC delivery efficiency across both cell lines (Fig. 3c). This result can be directly attributed to the greater hydrolytic stability conferred by molybdenum substitution. As established by ^183^W NMR spectroscopy (Fig. 2b), SiW_12_ undergoes extensive hydrolysis at near-physiological pH and is therefore less effective as a membrane carrier. SiMoW_11_, by contrast, retains greater structural integrity under the same conditions (Fig. 2a), ensuring that a higher proportion of the active, intact cluster is present at the membrane interface during the 2 h incubation period.

Both POMs facilitated phalloidin-TRITC uptake in HCT116 and HT29 cells with comparable concentration-dependent profiles (Fig. 3c). This similarity is notable given the substantial differences in lipid composition, membrane fluidity, and glycocalyx density between the two cell lines,^22–24^ suggesting that the superchaotropic transport mechanism operates robustly across distinct membrane environments. The superchaotropic membrane adsorption mechanism established for SiW_12_ and SiMoW_11_ in model membrane systems provides a working mechanistic framework for the cellular transport observed here.

## Conclusions

This work establishes that solution-phase integrity is a prerequisite for superchaotropic membrane-carrier activity, and that heterometal substitution is an effective and chemically precise strategy to enforce it. The superior performance of SiMoW_11_ over SiW_12_ demonstrates that single-atom composition changes at the addenda level translate directly into measurable differences in intracellular delivery efficiency, providing a clear design principle for the development of stability-optimized, all-inorganic POM carriers.

## Prof. Dr. Ulrich Schubert tribute

This work is dedicated to Prof. Dr. Ulrich Schubert (Technische Universität Wien) on the occasion of the themed collection honouring his 80th birthday. His pioneering contributions to inorganic materials chemistry and metal oxo cluster synthesis have profoundly shaped the field, and we are honoured to contribute to this collection celebrating his exceptional scientific legacy.

## Author contributions

NIG: conceptualization, data curation, formal analysis, funding acquisition, investigation, methodology, validation, visualization, writing – original draft; JB: investigation, formal analysis, methodology, validation, writing – review and editing; JR: investigation, formal analysis, writing – review and editing; GDF: conceptualization, funding acquisition, validation, writing – review and editing; AR: conceptualization, funding acquisition, project administration, writing – review and editing.

## Conflicts of interest

NIG and AR are inventors on a patent application related to this work filed by the University of Vienna (EP4483904A1). The authors declare that there are no other competing interests.

## Supplementary Material

TB-014-D6TB00818F-s001

## Data Availability

The dataset presented in this study is deposited in PHAIDRA repository: https://phaidra.univie.ac.at/o:2320540. Supplementary information (SI) is available. See DOI: https://doi.org/10.1039/d6tb00818f.
