# Supplementary material for: Polyoxometalates enable cellular uptake of the actin-binding toxin phalloidin
Source: J Mater Chem B. 2026 Aug 4;14(35):10836–42. doi: 10.1039/d6tb00818f (PMC13504987; doi:10.1039/d6tb00818f)
Supplement: Supplementary file 1 [file TB-014-D6TB00818F-s001.pdf]

Supplementary Information (SI) for Journal of Materials Chemistry B.
This journal is © The Royal Society of Chemistry 2026

Supporting Information

Polyoxometalates Enable Cellular Uptake of the Actin-Binding Toxin Phalloidin

Nadiia Gumerova,[a*] Janice Bergen,[b, c, d] Joshua Rieger,[a, d] Giorgia Del Favero,[b, c] and Annette Rompel[a, d*]

a. Universität Wien, Fakultät für Chemie, Institut für Biophysikalische Chemie, Josef-Holaubek-Platz 2, 1090 Wien, Austria, homepage: http://www.bpc.univie.ac.at.

E-mail: nadiia.gumerova@univie.ac.at, annette.rompel@univie.ac.at

b. Department of Food Chemistry and Toxicology, Faculty of Chemistry, University of Vienna, Vienna, Austria, homepage: https://lmc.univie.ac.at.

c. Core Facility Multimodal Imaging, Faculty of Chemistry, University of Vienna, Vienna, Austria, homepage: https://multimodal-imaging.univie.ac.at.

d. Universität Wien, Fakultät für Chemie, Vienna Doctoral School in Chemistry (DoSChem), Währinger Straße 42, 1090 Wien, Austria, homepage: https://doschem.univie.ac.at.

Table of Contents

1. Abbreviation list 2
2. Materials and Methods 2
3. Cell lines 3
4. Cell Viability 4
5. POM-Mediated Transport of Phalloidin-TRITC 6
6. NMR spectroscopic investigations 10
7. Isothermal titration calorimetry 15
References 16

1. Abbreviation list

ANOVA	analysis of variance	KRAS G13D	kirsten rat sarcoma viral proto-oncogene (KRAS), glycine-to-aspartate substitution at codon 13; an activating point mutation found in HCT116 colorectal carcinoma cells
ATCC	American type culture collection	LSD	least significant difference
BRAF V600E	B-Raf proto-oncogene serine/threonine kinase (*BRAF*), valine-to-glutamate substitution at codon 600; an activating point mutation found in HT29 colorectal carcinoma cells	MSI-H	microsatellite instable-high
β-CD	methyl-β-cyclodextrin	MSS	microsatellite stable
DAPI	4′,6-diamidino-2-phenylindole	NMR	nuclear magnetic resonance
DLS	dynamic light scattering	NIBS	non-invasive backscatter
DMEM	Dulbecco's modified eagle medium	PBS	phosphate buffered saline
DMSO	dimethyl sulfoxide	POM	polyoxometalate
DSMZ	Deutsche Sammlung von Mikroorganismen und Zellkulturen (German collection of microorganisms and cell cultures)	r.f.u.	relative fluorescence units
DP	differential power	Tris	tris(hydroxymethyl)aminomethan
ELISA	enzyme-linked immunosorbent assay	TRITC	5(6)-tetramethylrhodaminisothiocyanat
IR spectroscopy	infrared spectroscopy	SiW_{12}	$K_4[\alpha\text{-}Si^{IV}W^{VI}_{12}O_{40}]\cdot 14H_2O$
ITC	isothermal titration calorimetry	$SiMoW_{11}$	$K_4[\alpha\text{-}Si^{IV}Mo^{VI}W^{VI}_{11}O_{40}]\cdot 24H_2O$

2. Materials and Methods

The cell culture plasticware was purchased at Sarstedt AG & Co (Nümbrecht, Germany) and microscopy plastics and glassware at ibidi (Graefeling, Germany). The cell growth media (McCoy's 5a – composition could be find here https://www.thermofisher.com/order/catalog/product/26600080, Dulbecco's Modified Eagle – composition could be find here https://www.thermofisher.com/order/catalog/product/26600080) and supplements (fetal calf serum, penicillin/streptomycin, amino acids, phenol red, Hoechst dye) were bought from Gibco Thermo Fisher Scientific (Waltham, MA, USA) and Sigma-Aldrich Chemie GmbH & Co

(Steinheim, Germany). The CellTiter-Blue (10×) concentrate was obtained from Promega (Waldorf, Germany). Phalloidin-TRITC was purchased from Sigma-Aldrich (Austria). $Na_2WO_4 \cdot 2H_2O$ (≥99%, ACS reagent), $Na_2MoO_4 \cdot 2H_2O$ (≥99%, ACS reagent), Na_2SiO_3 (reagent grade), KCl (≥99%, ACS reagent), and HNO_3 (63%, ACS reagent) were purchased from Sigma-Aldrich (Austria) and used without further purification.

The polyoxometalate (POM) salts $K_4[\alpha\text{-}Si^{IV}W^{VI}_{12}O_{40}]\cdot 14H_2O$ (**SiW_{12}**) [1] and $K_4[\alpha\text{-}Si^{IV}Mo^{VI}W^{VI}_{11}O_{40}]\cdot 24H_2O$ (**$SiMoW_{11}$**) [2] were prepared according to reported procedures [1, 2]. The samples used in this study originate from the same synthetic batch as reported previously in ref. [3]. The detailed characterization of **SiW_{12} and $SiMoW_{11}$**, including NMR spectroscopic data, infrared (IR) spectroscopic data, and thermogravimetric analysis (TGA) data, has been described in [3].

Dynamic light scattering (DLS)

The measurements were performed at 25 °C using a Malvern Zetasizer Nano and a PCS1115 glass cuvette. The data were acquired in 173° backscatter mode (NIBS) with automatic attenuation. The samples were diluted 1:50 in 10 mM phosphate buffer pH 7.4 to a final volume of 1.0 mL. For each sample, three independently prepared solutions were measured; for each solution, four consecutive measurement cycles were recorded, each comprising 12 runs of 20 s. Size and polydispersity index values are reported as the mean of the three independent measurements.

Isothermal titration calorimetry (ITC).

All experiments were performed with a PEAQ-ITC Automated (Malvern, Microcal) at atmospheric pressure and 298.15 K, with reference power of 10 µcal/sec, and stir speed of 750 rpm. A constant volume of $[SiMoW_{11}O]^{4-}$ (2 µL per injection) was injected into the phalloidin solution (5 mM stock in DMSO has been diluted with 10 mM Tris, pH 7.4 to the 10 µM concentration) to determine the apparent binding affinity. Dilution heats were determined by titration of the POM into buffer and subtracted from the reaction heat. Evaluation software: Microcal PEAQ-ITC Analysis Software v1.22.

3. Cell lines

The HCT116 cell line was acquired from ATCC and maintained in McCoy's 5a medium supplemented with 10% fetal calf serum and 1% penicillin/streptomycin. The HT29 cell line was obtained from the German Collection of Microorganisms and Cell Cultures (DSMZ, Braunschweig, Germany) and maintained in Dulbecco's Modified Eagle Medium supplemented with 10% fetal calf serum, 1% penicillin/streptomycin, 1% non-essential amino acids and 1% L-glutamine. Both cell lines were kept in an incubator at 37 °C (5% CO_2) and passaged two to three times per week after reaching a cell confluency of approximately 80 % [4].

Table S1. Comparative properties of the HCT116 and HT29 human colorectal carcinoma cell lines. Molecular and membrane-compositional data are derived from published literature [5, 6]. MSI-H, microsatellite instable-high; MSS, microsatellite stable.

Property	HCT116	HT29

Property	HCT116	HT29
**Molecular profile**		
Microsatellite status	**MSI-H** (instable)	**MSS** (stable)
Driver mutation	*KRAS* G13D	*BRAF* V600E
Differentiation state	undifferentiated epithelial; uniform morphology	partial enterocytic differentiation; heterogeneous
**Membrane composition**		
Principal lipids	phosphatidylcholine and phosphatidylethanolamine; moderate cholesterol	elevated sphingomyelin and glycosphingolipids; higher cholesterol
Membrane fluidity	**high** — fluid, dynamic bilayer	**low** — rigid bilayer
Glycocalyx	thin; low mucin expression	thick; heavily O-glycosylated mucins (Mucin 5AC, Mucin 2)
**Endocytic activity**		
Clathrin-mediated endocytosis	**active**	**reduced**
Macropinocytosis	**active**	**reduced**
**Expected POM transport behavior**		
POM membrane adsorption	**favored** — fluid membrane permits POM interaction	**hindered** — rigid membrane and glycocalyx barrier
Phalloidin-TRITC uptake (this work)	**more pronounced**	**attenuated**

4. Cell Viability

Cells were seeded with the culture medium at a density of 1.09×10^5 cells/cm² for HCT116 and 1.25×10^5 cells/cm² for HT29 in complete culture medium (based on McCoy's 5a or DMEM) and allowed to adhere for 24 h. The medium was then replaced with **SiW_{12}** or **$SiMoW_{11}$** solutions prepared in serum-free medium at the indicated concentrations, and cells were incubated for a further 24 h. Subsequently, the medium was aspirated and a 1:10 dilution of CellTiter-Blue reagent (Promega) in phenol red-free DMEM was added to each well for 2 h at 37 °C (5% CO_2). The supernatant was transferred to a black medium-binding 96-well ELISA plate (REF: 82.1581.120, Sarstedt AG & Co, Nümbrecht, Germany) and fluorescence was measured at 560/590 nm using a Synergy H1 Hybrid multi-detection microplate reader (BioTek, Bad Friedrichshall, Germany).

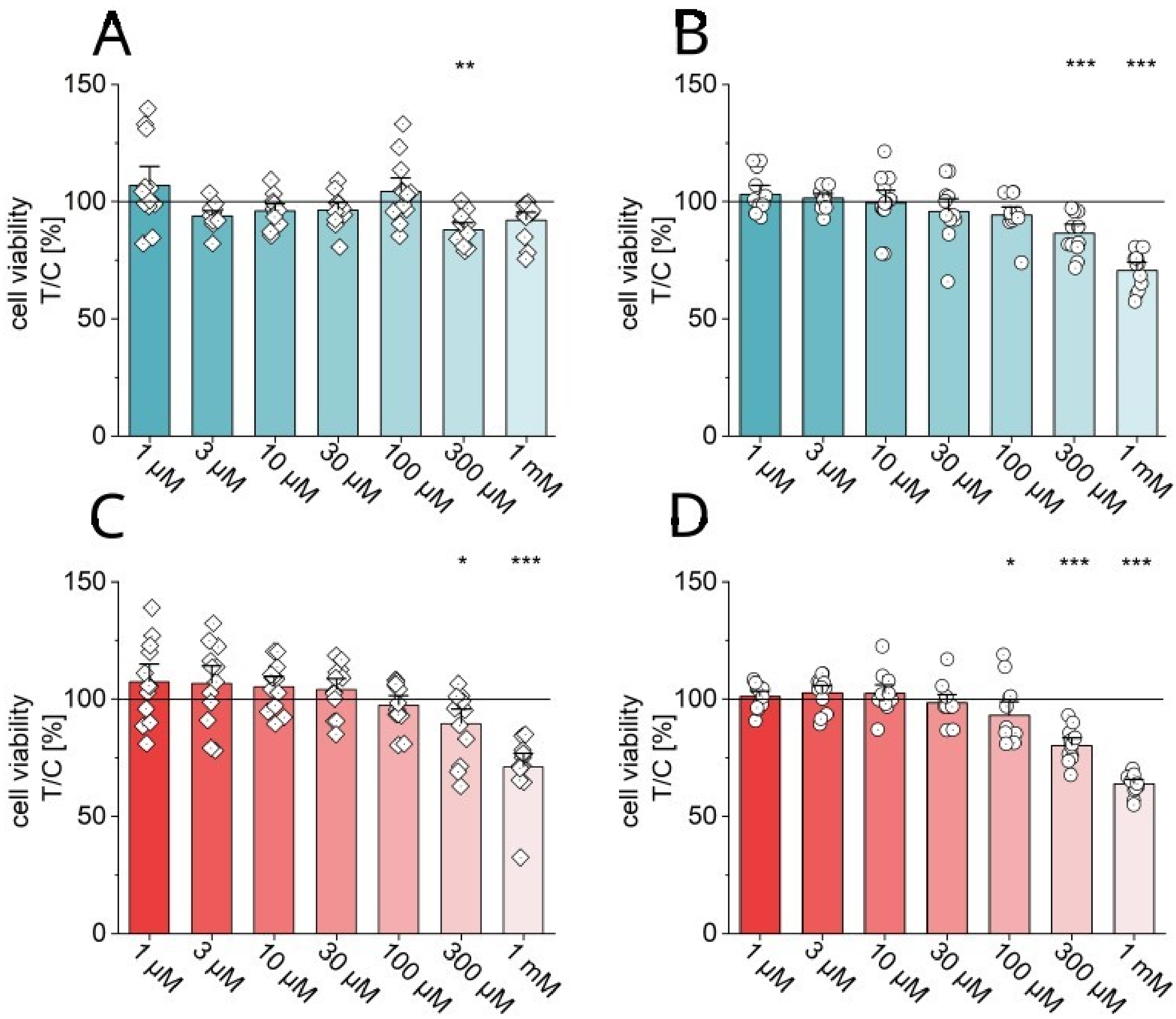

Figure S1. Cytotoxic impact of **SiW_{12}** (A, B) and **$SiMoW_{11}$** (C, D) on HCT116 (A, C) and HT29 (B, D) tested after 24 hours incubation with POMs. Statistical differences between groups were assessed by one-way ANOVA followed by Fisher's Least Significant Difference (LSD) post-hoc test for pairwise comparisons and are marked * ($p<0.05$), ** ($p<0.01$), *** ($p<0.001$). The experiments were performed on $n \geq 3$ cell preparations and measured in technical triplicates. The cell viability is expressed as the percentage of the untreated control (T/C, %), where T/C = (mean fluorescence of treated cells / mean fluorescence of untreated control cells) × 100.

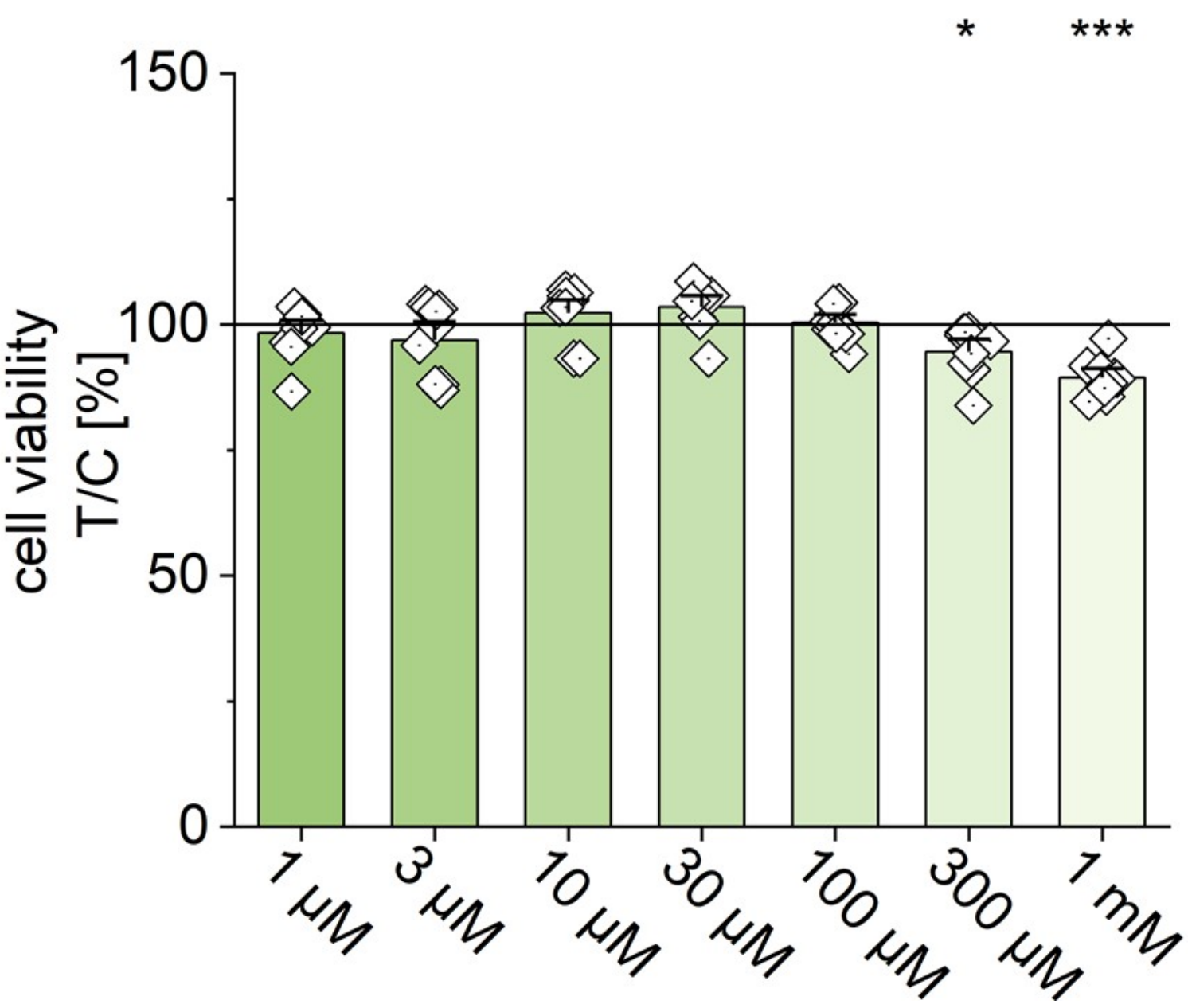

Figure S2. Cytotoxic impact of **SiMoW$_{11}$** (1 µM – 1 mM) and 2.5 µM phalloidin on HCT116 after 2 h hours incubation. Statistical differences between groups were assessed by one-way ANOVA followed by Fisher's Least Significant Difference (LSD) post-hoc test for pairwise comparisons and are marked * (p<0.05), *** (p<0.001). The experiments were performed on n ≥ 3 cell preparations and measured in technical triplicates. The cell viability is expressed as the percentage of the untreated control (T/C, %), where T/C = (mean fluorescence of treated cells / mean fluorescence of untreated control cells) × 100.

5. POM-Mediated Transport of Phalloidin-TRITC

HCT116 and HT29 cells were seeded in 8-well µ-dishes (ibidi, Gräfelfing, Germany) at a density of 120,000 cells per well and allowed to adhere and grow for 24 h under standard culture conditions (McCoy's 5A medium supplemented with 10% fetal bovine serum and 1% penicillin/streptomycin for HCT116; DMEM supplemented with 10% fetal bovine serum and 1% penicillin/streptomycin for HT29; 37 °C, 5% CO_2). For the uptake experiments, 1 mM aqueous stock solutions of **SiMoW$_{11}$** or **SiW$_{12}$** were diluted in serum-free culture medium to final concentrations of 10, 25, and 50 µM (**SiMoW$_{11}$**) or 50 µM (**SiW$_{12}$**), each supplemented with 2.5 µM phalloidin-TRITC (added from a DMSO 0.5 mM stock) and Hoechst 33342 (1.25 µL per well). Hoechst 33342 is a cell-permeable fluorescent dye that binds selectively to double-stranded DNA and stains cell nuclei blue, enabling unambiguous identification of individual cells and assessment of nuclear morphology independently of the F-actin channel. These solutions were applied to the adherent cell monolayers and incubated for 2 h at 37 °C (5% CO_2). After incubation, cells were washed twice with PBS to remove unbound material and fixed with 4% formaldehyde in PBS for 15 min at room temperature. The fixed samples were mounted in Fluoroshield mounting medium without DAPI (Abcam, Cambridge, UK). The widefield fluorescence images were acquired using a BioTek Lionheart FX automated microscope equipped with a Texas Red filter cube (excitation 562/40 nm, emission 624/40 nm) for phalloidin-TRITC detection and a DAPI filter cube (excitation 377/50 nm, emission 447/60 nm) for Hoechst 33342 nuclear staining. The fluorescence intensity was quantified in defined

regions of interest across multiple fields of view, and mean intensity values per field were used for statistical comparison between conditions. High-resolution confocal images for cytoskeletal morphology assessment were acquired on a Zeiss LSM 800 confocal microscope equipped with a 63× oil immersion objective, using a 561 nm laser line for phalloidin-TRITC detection and a 405 nm laser line for Hoechst 33342. For graphical representation, the phalloidin-TRITC signal is depicted in green and the nuclear Hoechst 33342 signal in blue.

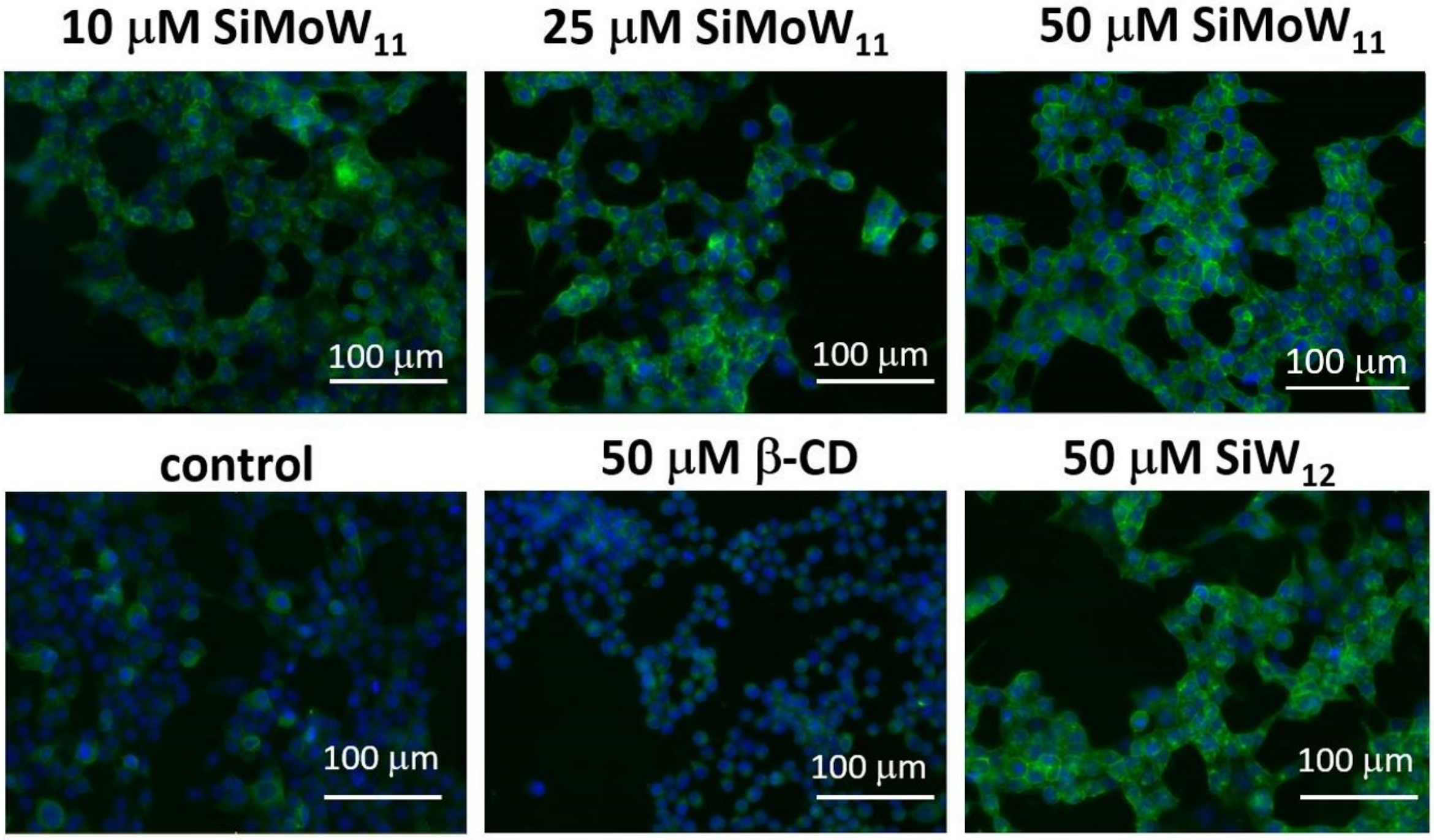

Figure S3. Fluorescence microscopy. HCT116 cells were incubated for 2 h with Phalloidin-TRITC (green) in the presence or absence of two POMs – $\mathbf{SiMoW_{11}}$ and $\mathbf{SiW_{12}}$, and methyl-β-cyclodextrin (β-CD). Cells treated with phalloidin-TRITC alone served as the negative control, confirming the membrane impermeability of phalloidin in the absence of a carrier. Cell nuclei are counterstained with Hoechst 33342 (blue).

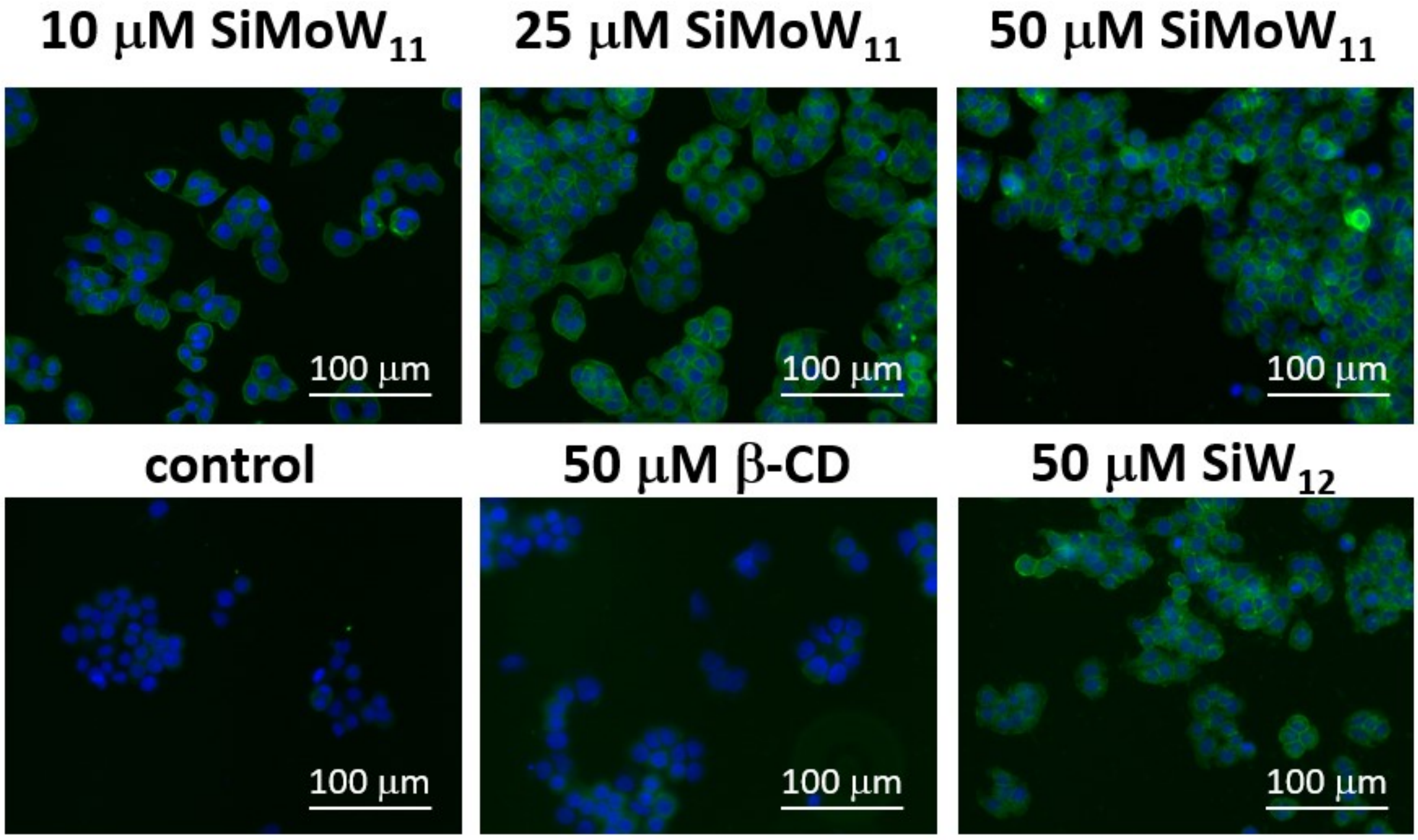

Figure S4. Fluorescence microscopy. HT29 cells were incubated for 2 h with Phalloidin-TRITC (green) in the presence or absence of two POMs – $\mathbf{SiMoW_{11}}$ and $\mathbf{SiW_{12}}$, and methyl-β-cyclodextrin (β-CD). Cells treated with phalloidin-TRITC alone served as the negative control,

confirming the membrane impermeability of phalloidin in the absence of a carrier. Cell nuclei are counterstained with Hoechst 33342 (blue).

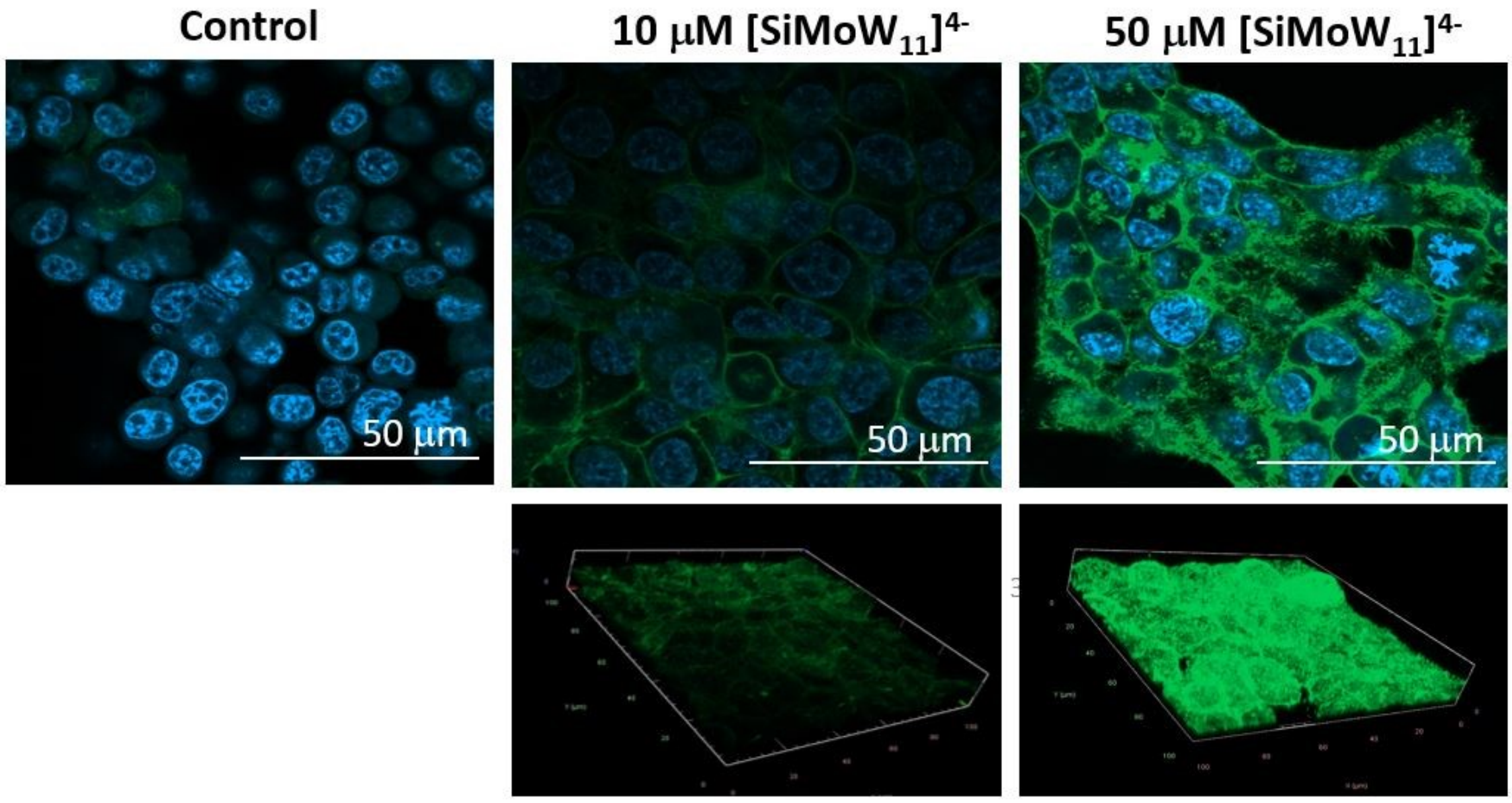

Figure S5. **Confocal microscopy images of phalloidin-TRITC uptake in HCT116 cells**. The cells were incubated with phalloidin-TRITC (2.5 μM) in the presence or absence of $\mathbf{SiMoW_{11}}$ for 2 h, fixed, and imaged on a Zeiss LSM 800 confocal laser scanning microscope using a 63× oil immersion objective. The single optical sections (xy-plane) are shown alongside three-dimensional reconstructions generated from z-stacks, spanning the full cell volume. Z-stack images were processed and rendered using Fiji software. The intracellular phalloidin-TRITC fluorescence is shown in green and the cell nuclei counterstained with Hoechst 33342 are shown in blue. Scale bar: 50 μm.

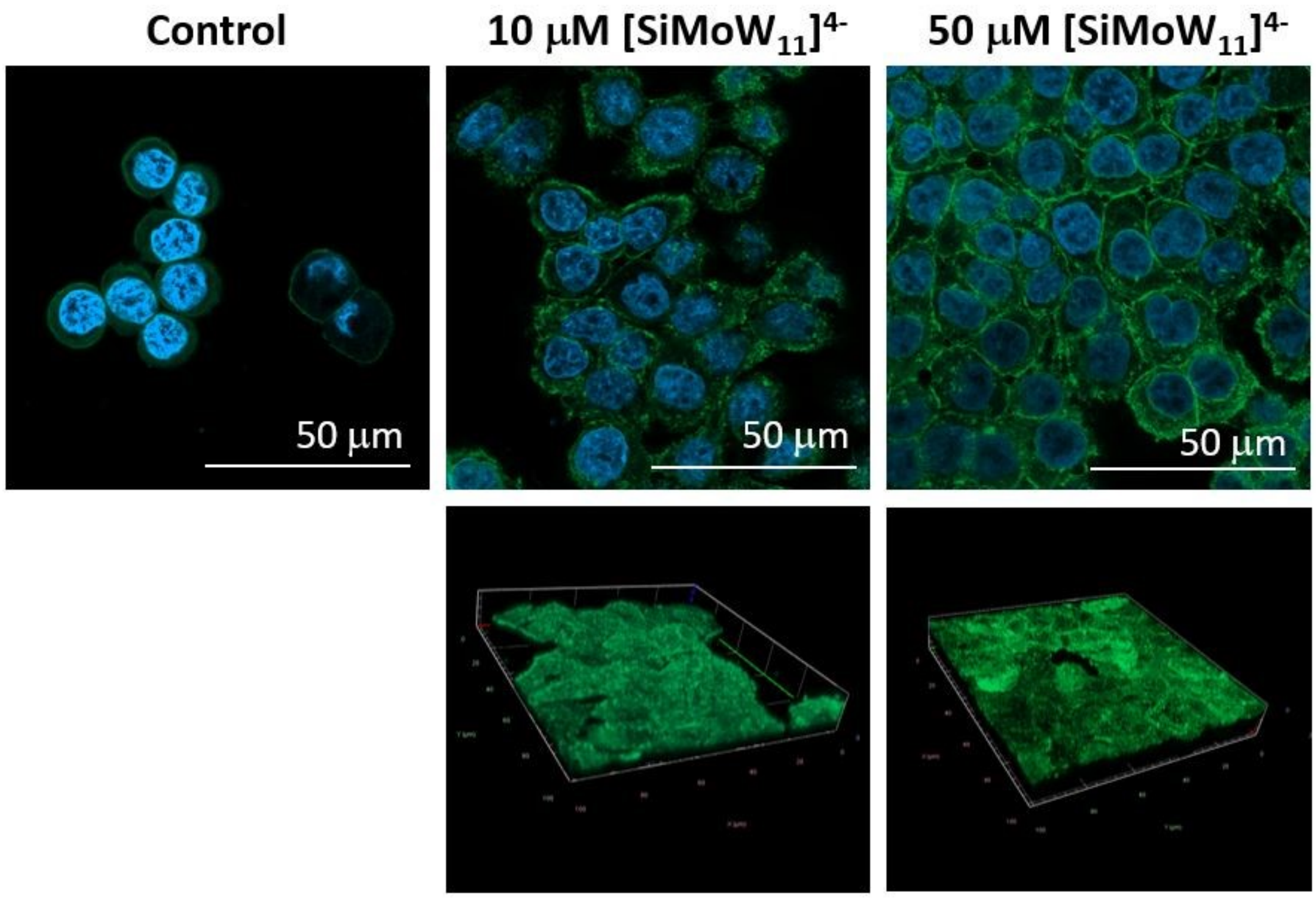

Figure S6. Confocal microscopy images of phalloidin-TRITC uptake in HT29 cells. The cells were incubated with phalloidin-TRITC (2.5 µM) in the presence or absence of **$SiMoW_{11}$** for 2 h, fixed, and imaged on a Zeiss LSM 800 confocal laser scanning microscope using a 63× oil immersion objective. Single optical sections (xy-plane) are shown alongside three-dimensional reconstructions generated from z-stacks, spanning the full cell volume. The Z-stack images were processed and rendered using Fiji software. The intracellular phalloidin-TRITC fluorescence is shown in green and the cell nuclei counterstained with Hoechst 33342 are shown in blue. Scale bar: 50 µm.

6. NMR spectroscopic investigations

^{1}H and ^{183}W NMR spectra were recorded on an Avance Neo500-MHz FT-NMR spectrometer (Bruker, Rheinstetten, Germany) at 25°C. The measurements were performed at the NMR center, Faculty of Chemistry, University of Vienna. The chemical shifts (δ) are reported in ppm and referenced to the residual solvent signal (HDO in D_2O, δ = 4.79 ppm) for ^{1}H and to 1 M Na_2WO_4 for ^{183}W. For ^{183}W NMR analysis, 2.7 mL samples of a 60 mg/mL aqueous POM solutions were measured in 10 mm NMR tubes. The experimental time was about 60 hours, using a standard pulse program at 20.836 MHz, with a flip angle of 63° and a 1 s relaxation delay.

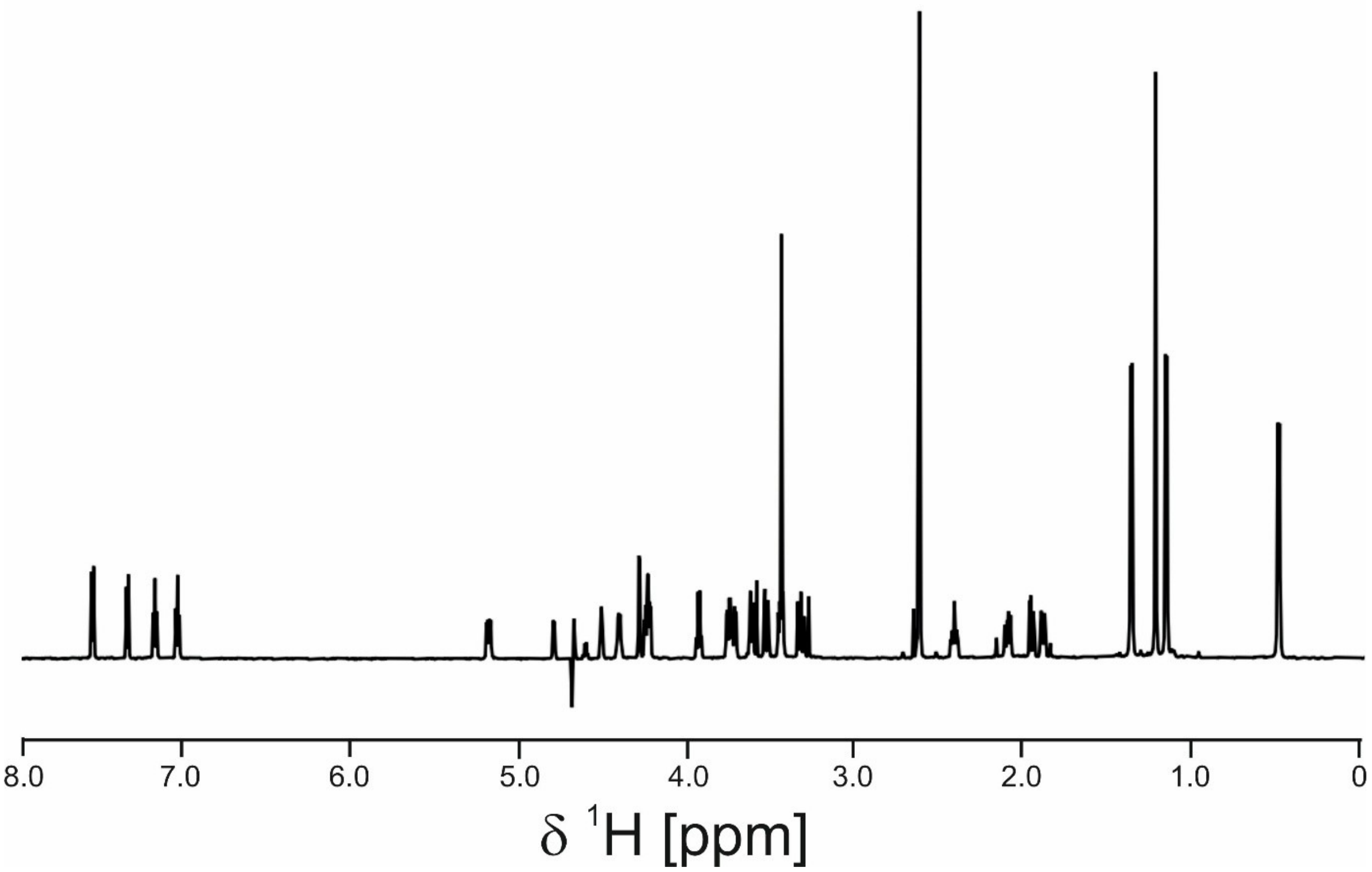

Figure S7. ^{1}H NMR spectrum of 0.5 mM phalloidin (stock in DMSO-d_6) in D_2O with 150 mM NaCl. ^{1}H NMR (700 MHz, D_2O) δ [ppm]: 7.58, 7.57, 7.38, 7.36, 7.22, 7.21, 7.20, 7.08, 7.07, 7.06, 5.23, 5.22, 5.21, 5.20, 4.83, 4.83, 4.82, 4.82, 4.71, 4.65, 4.64, 4.63, 4.62, 4.54, 4.54, 4.45, 4.45, 4.44, 4.44, 4.43, 4.42, 4.32, 4.32, 4.29, 4.28, 4.27, 4.27, 4.26, 4.26, 4.26, 4.25, 3.98, 3.97, 3.96, 3.95, 3.80, 3.79, 3.78, 3.78, 3.77, 3.76, 3.75, 3.74, 3.66, 3.64, 3.62, 3.57, 3.57, 3.55, 3.55, 3.49, 3.48, 3.47, 3.46, 3.37, 3.35, 3.35, 3.33, 3.31, 2.74, 2.68, 2.67, 2.66, 2.65, 2.65, 2.65, 2.64,

2.64, 2.55, 2.46, 2.45, 2.44, 2.44, 2.43, 2.42, 2.42, 2.19, 2.14, 2.12, 2.12, 2.10, 1.99, 1.98, 1.97, 1.96, 1.92, 1.90, 1.87, 1.39, 1.38, 1.24, 1.18, 1.17, 1.14, 1.13, 0.98, 0.51, 0.50.

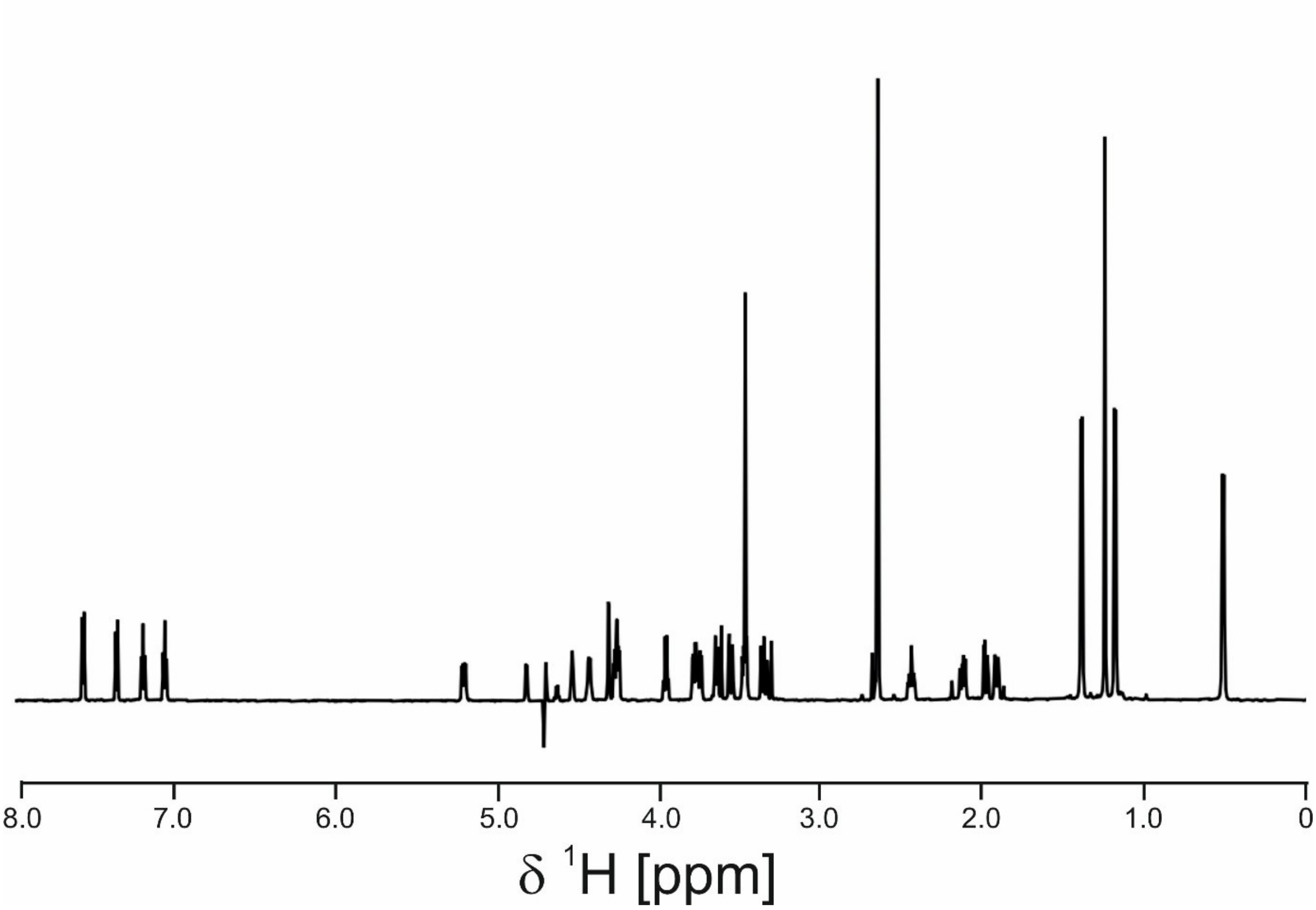

Figure S8. ^{1}H NMR spectrum of 0.5 mM phalloidin (stock in DMSO-d_6) and 0.5 mM of **$SiMoW_{11}$** in D_2O with 150 mM NaCl. ^{1}H NMR (700 MHz, D_2O) δ 7.59, 7.58, 7.38, 7.37, 7.22, 7.21, 7.20, 7.09, 7.08, 7.07, 5.22, 5.21, 5.20, 5.20, 4.84, 4.84, 4.83, 4.83, 4.72, 4.72, 4.71, 4.65, 4.64, 4.63, 4.62, 4.56, 4.55, 4.55, 4.54, 4.45, 4.45, 4.44, 4.44, 4.43, 4.43, 4.42, 4.42, 4.32, 4.31, 4.29, 4.29, 4.28, 4.27, 4.26, 4.26, 4.25, 3.98, 3.97, 3.96, 3.95, 3.81, 3.80, 3.79, 3.79, 3.77, 3.76, 3.75, 3.74, 3.67, 3.65, 3.62, 3.59, 3.58, 3.56, 3.56, 3.49, 3.48, 3.48, 3.47, 3.46, 3.38, 3.36, 3.35, 3.34, 3.31, 2.75, 2.68, 2.65, 2.65, 2.65, 2.65, 2.64, 2.55, 2.47, 2.46, 2.45, 2.45, 2.44, 2.43, 2.43, 2.19, 2.14, 2.13, 2.12, 2.11, 2.00, 1.99, 1.97, 1.97, 1.93, 1.90, 1.87, 1.39, 1.38, 1.24, 1.18, 1.17, 1.14, 1.13, 0.99, 0.52, 0.51.

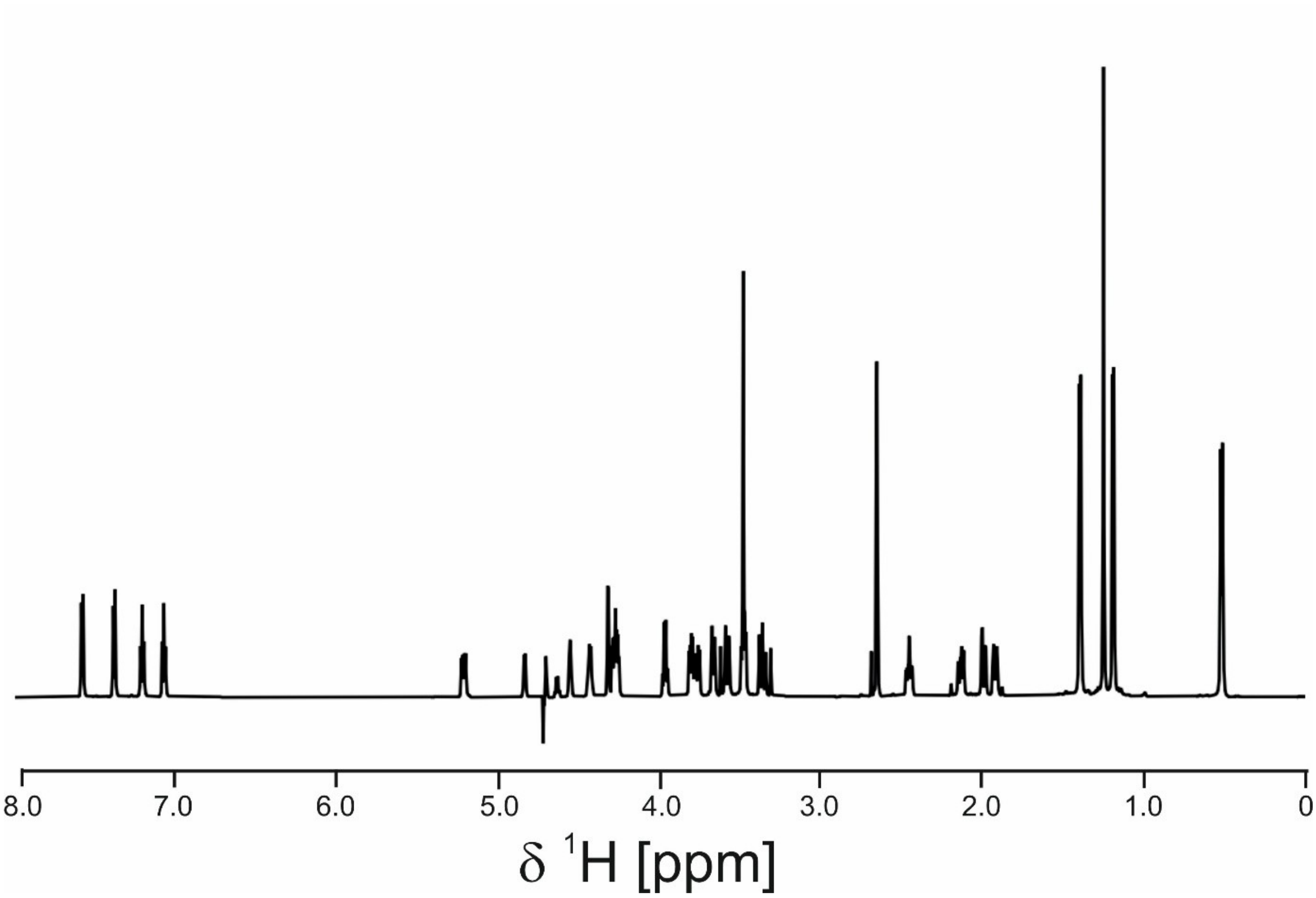

Figure S9. ^{1}H NMR spectrum of 0.5 mM phalloidin (stock in DMSO-d_6) and 1 mM of **$SiMoW_{11}$** in D_2O with 150 mM NaCl. ^{1}H NMR (700 MHz, D_2O) δ 7.59, 7.58, 7.39, 7.38, 7.22, 7.21, 7.20, 7.09, 7.08, 7.07, 5.23, 5.22, 5.21, 5.20, 4.84, 4.83, 4.83, 4.70, 4.65, 4.64, 4.63, 4.62, 4.56, 4.55, 4.45, 4.44, 4.43, 4.43, 4.42, 4.42, 4.41, 4.32, 4.32, 4.29, 4.29, 4.28, 4.27, 4.26, 4.26, 4.25, 3.98, 3.97, 3.96, 3.95, 3.82, 3.81, 3.80, 3.79, 3.78, 3.77, 3.76, 3.75, 3.67, 3.66, 3.62, 3.59, 3.59, 3.57, 3.57, 3.49, 3.48, 3.47, 3.46, 3.38, 3.36, 3.36, 3.34, 3.31, 2.68, 2.65, 2.65, 2.65, 2.65, 2.64, 2.47, 2.46, 2.46, 2.45, 2.44, 2.44, 2.43, 2.19, 2.15, 2.13, 2.12, 2.11, 2.00, 1.99, 1.98, 1.97, 1.93, 1.92, 1.91, 1.87, 1.48, 1.39, 1.38, 1.34, 1.28, 1.24, 1.19, 1.18, 1.15, 1.13, 0.98, 0.51, 0.50.

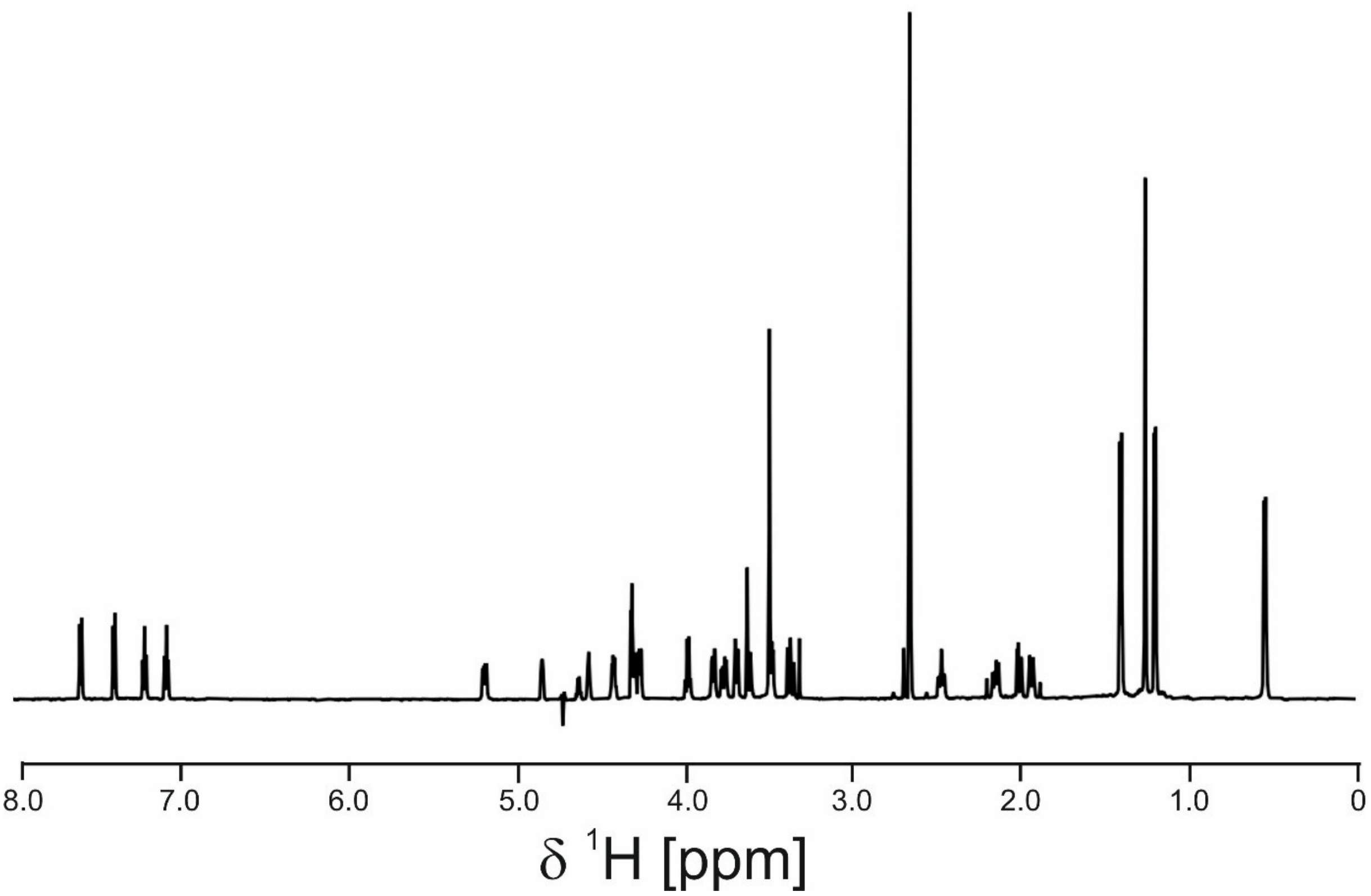

Figure S10. ^{1}H NMR spectrum of 0.5 mM phalloidin (stock in DMSO-d_6) and 1.5 mM of **$SiMoW_{11}$** in D_2O with 150 mM NaCl. ^{1}H NMR (700 MHz, D_2O) δ 7.60, 7.59, 7.41, 7.39, 7.23, 7.22, 7.21, 7.10, 7.09, 7.08, 5.20, 5.19, 5.18, 5.18, 4.85, 4.85, 4.84, 4.84, 4.73, 4.72, 4.71, 4.64, 4.63, 4.62, 4.61, 4.57, 4.44, 4.43, 4.42, 4.42, 4.41, 4.41, 4.32, 4.31, 4.30, 4.29, 4.29, 4.27, 4.27, 4.26, 4.25, 3.99, 3.98, 3.97, 3.96, 3.83, 3.83, 3.82, 3.81, 3.78, 3.77, 3.76, 3.75, 3.69, 3.68, 3.62, 3.60, 3.60, 3.49, 3.47, 3.47, 3.38, 3.36, 3.36, 3.34, 3.31, 2.75, 2.69, 2.66, 2.66, 2.65, 2.65, 2.65, 2.55, 2.48, 2.48, 2.47, 2.46, 2.46, 2.45, 2.44, 2.19, 2.16, 2.14, 2.13, 2.12, 2.01, 2.01, 1.99, 1.98, 1.94, 1.92, 1.87, 1.40, 1.39, 1.28, 1.25, 1.19, 1.18, 1.14, 0.54, 0.53.

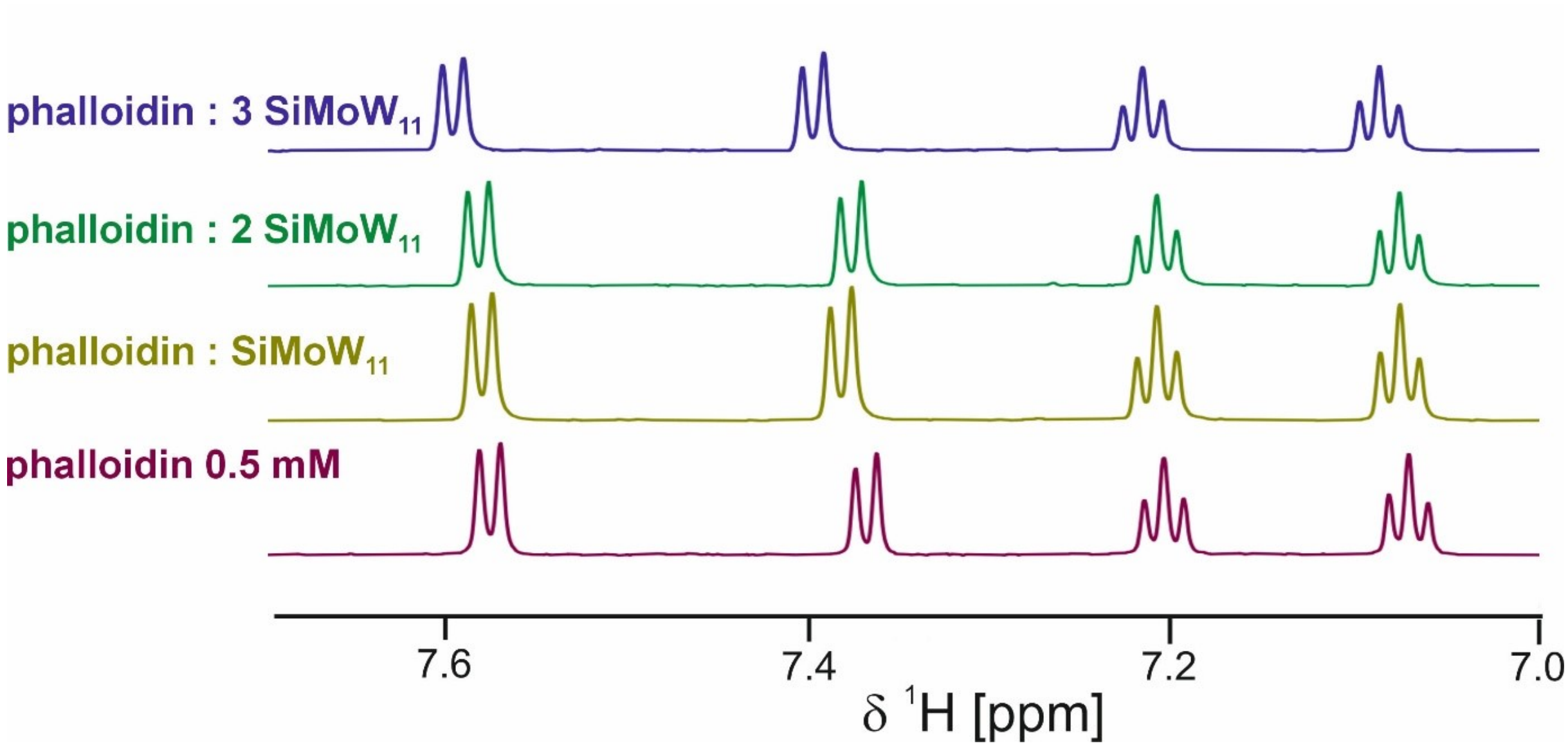

Figure S11. The aromatic region of phalloidin in ^1H NMR spectra of 0.5 mM phalloidin (stock in DMSO-d_6) and 0-1.5 mM of **$SiMoW_{11}$** in D_2O with 150 mM NaCl.

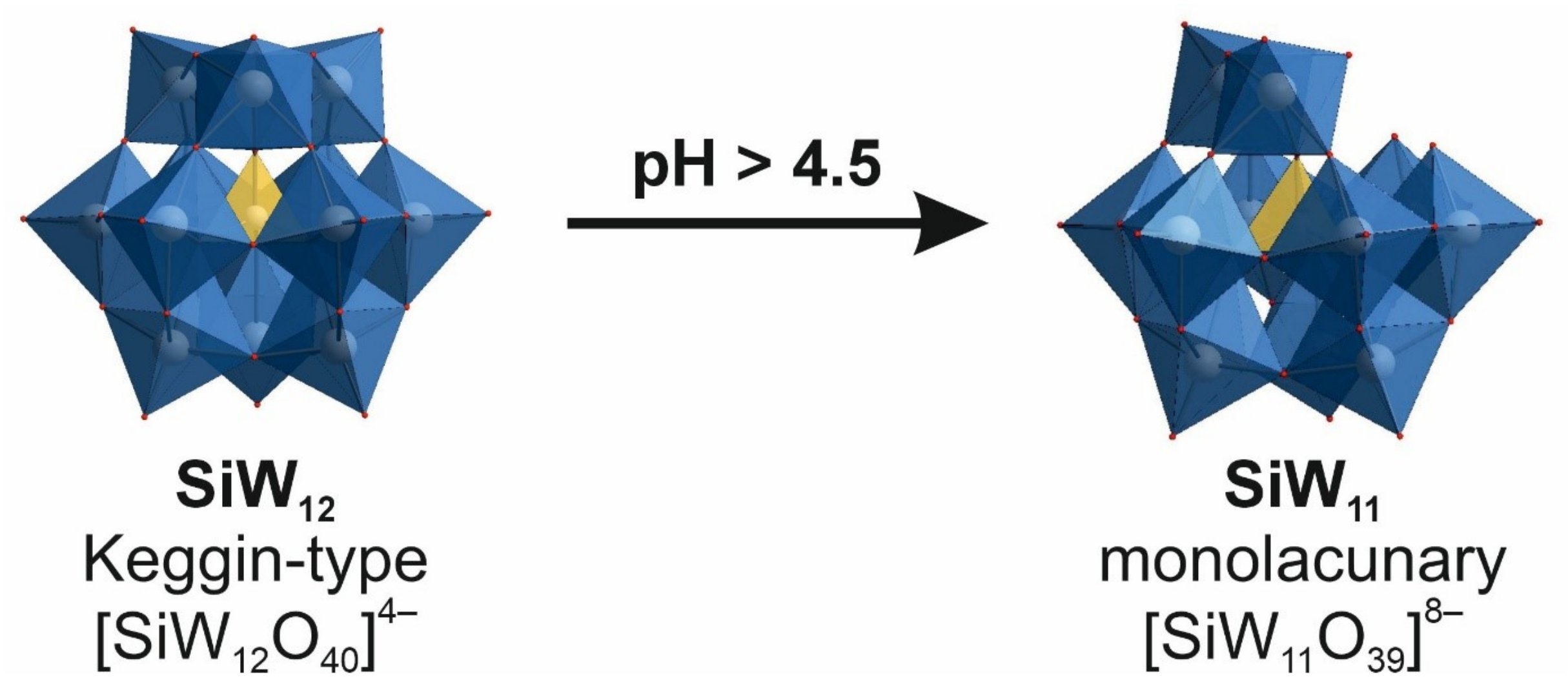

Scheme S1. Hydrolysis of **SiW_{12}** to the monolacunary species $[SiW_{11}O_{39}]^{8-}$ under aqueous buffered conditions. Loss of one {WO} unit from the intact Keggin framework generates the C*s*-symmetric lacunary anion, accompanied by an increase in formal charge from 4− to 8−. This hydrolysis is promoted by near neutral to mildly alkaline pH and is significantly accelerated in the presence of buffering agents, as demonstrated by ^{183}W NMR spectroscopy (Figure 2, main manuscript). Color code: $\{WO_6\}$, blue; $\{SiO_4\}$, yellow; O, red.

7. Isothermal titration calorimetry

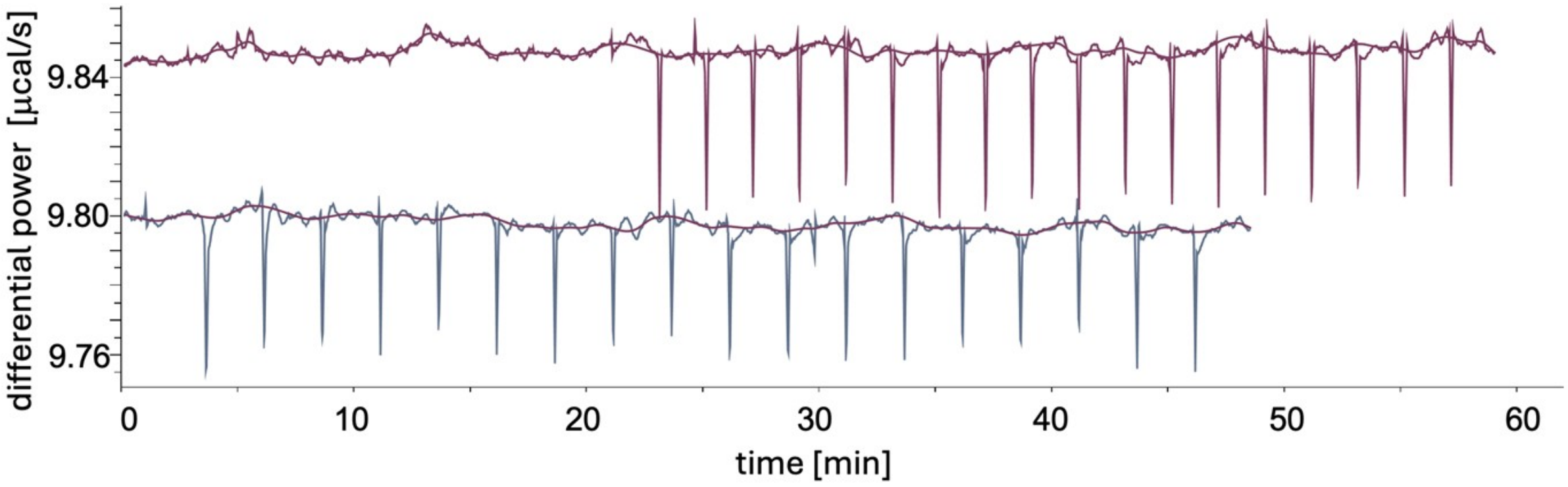

Figure S12. Microcalorimetric titrations of **SiMoW$_{11}$** with phalloidin in 10 mM Tris-HCl buffer (pH 7.4, 0.2% DMSO) at 25 °C. Raw thermogram showing differential power (DP) as a function of time for the titration of **SiMoW$_{11}$** (200 µM, syringe) into phalloidin (10 µM, cell) over 19 injections of 2 µL each (pink), overlaid with a water–water reference control (blue). The absence of any differential heat signal above the baseline, and the near-identical appearance of the experimental and control thermograms, confirm that no detectable binding interaction occurs between **SiMoW$_{11}$** and phalloidin under these conditions.

References

1. G. H. A. Téazéa, R. G. Finke and D. K. Lyon, in *Inorganic Syntheses*, ed. A. P. Ginsberg, Wiley, New York, 1990, vol. **27**, pp. 85–96.
2. C. Sanchez, J. Livage, J. P. Launay, M. Fournier and Y. Jeannin, *J. Am. Chem. Soc.*, 1982, **104**, 3194–3202, doi: 10.1021/ja00375a044.
3. A. Barba-Bon, N. I. Gumerova, E. Tanuhadi, M. Ashjari, Y. Chen, A. Rompel and W. M. Nau, *Adv. Mater.*, 2024, **36**, 2309219, doi: 10.1002/adma.202309219.
4. G. Del Favero, M. Zeugswetter, E. Kiss and D. Marko, *Front. Pharmacol.*, 2021, **12**, 647350, doi: 10.3389/fphar.2021.647350.
5. D. Ahmed, P. W. Eide, I. A. Eilertsen, S. A. Danielsen, M. Eknæs, M. Hektoen, G. E. Lind and R. A. Lothe, *Oncogenesis*, 2013, **2**, e71–e71, doi: 10.1038/oncsis.2013.35.
6. J. Fogh and G. Trempe, in *Human Tumor Cells in Vitro*, ed. J. Fogh, Springer US, Boston, MA, 1975, doi: 10.1007/978-1-4757-1647-4_5, pp. 115–159.
